# How to Track Mechanochemical Reactions in Resonant Acoustic Mixers by in Situ Raman Spectroscopy

**DOI:** 10.1002/chem.202501336

**Published:** 2025-08-08

**Authors:** Steffi Krause Hinojosa, Tugce Dogan, Sven Fabig, Sven Grätz, Lars Borchardt

**Affiliations:** ^1^ Inorganic Chemistry I Ruhr‐University Bochum Universitätstraße 150 Bochum 44801 Germany

**Keywords:** catalysis reaction, condensation reactions, in situ Raman, RAM, ZIF

## Abstract

Mechanochemical processes have traditionally been explored using ball mills; however, resonant acoustic mixers (RAM) offer a compelling alternative, operating without milling media and enabling scalable, solvent‐free synthesis. Despite their advantages, direct transfer of reaction conditions from ball mills to RAM has proven nontrivial, requiring careful optimization of key parameters. In this study, we systematically define an operational window for effective in situ Raman monitoring in RAM. Critical parameters—including vessel material, applied gravitational force, filling degree, substrate rheology, and reaction time—were assessed to ensure high‐quality spectral acquisition. Utilizing a sapphire‐glass window and a precisely aligned laser setup, we achieved strong, reproducible Raman signals under controlled conditions. The optimal window was found to include a minimum filling degree of 10% and applied g‐forces between 20 and 70 g. This framework was validated across diverse mechanochemical reactions, including organic condensations (Knoevenagel and quinoxaline), metal‐organic framework formation (ZIF), and catalytic coupling (Glaser reaction), demonstrating the robustness and versatility of in situ Raman spectroscopy in RAM‐based synthesis.

## Graphical abstract

We define a practical operational window for in situ Raman monitoring in resonant acoustic mixing (RAM), a milling media‐free alternative to ball milling. By optimizing key parameters, including vessel design, filling degree, and acceleration, we establish conditions that enable robust spectral acquisition across various mechanochemical transformations. This framework facilitates reliable reaction tracking in RAM.

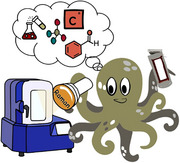

## 1 Introduction

Mechanochemistry is transforming synthetic strategies by replacing traditional solvents and harsh conditions with mechanical energy, offering a more sustainable and efficient route for chemical transformations. According to the International Union of Pure and Applied Chemistry (IUPAC), mechanochemistry is defined as a chemical reaction induced by the direct absorption of mechanical energy.^[^ ^1^ ^]^ As an interdisciplinary field, mechanochemistry encompasses various branches of chemistry, including material science,^[^ ^2^, ^3^, ^4^ ^]^ polymer chemistry,^[^ ^5^, ^6^ ^]^ organic,^[^ ^7^, ^8^, ^9^ ^]^ and inorganic chemistry.^[^ ^4^, ^10^, ^11^ ^]^ Its key advantages include solvent‐free conditions, environmental compatibility, energy efficiency, and enhanced safety over traditional solution‐based methods.^[^ ^12^, ^13^ ^]^ To facilitate mechanochemical reactions, a variety of specialized devices have been developed to perform under different reaction conditions. Traditionally, mechanochemical transformations are performed using ball mills, where reactions are initiated by collisions between milling balls. More recently, Resonant Acoustic Mixers (RAM) have emerged as a promising alternative.^[^ ^14^, ^15^, ^16^ ^]^ Unlike ball mills, RAM operates without milling media, under milder conditions, and at a fixed mechanical resonance frequency (∼60 Hz) with tuneable gravitational acceleration (10–100 g). RAM delivers vibrational energy through a spring‐mounted plate, generating local mixing zones that minimize contamination and facilitate scale‐up.^[^ ^15^, ^16^ ^]^ RAM has already been successfully applied to synthesize metal‐organic frameworks (MOFs),^[^ ^17^ ^]^ cocrystals,^[^ ^14^ ^]^ and catalytic reactions.^[^ ^15^, ^18^ ^]^ While RAM is relatively new in mechanochemical synthesis, vibrational energy has long been employed in industrial applications such as battery electrode fabrication,^[^ ^19^ ^]^ energetic materials processing,^[^ ^20^ ^]^ and pharmaceutical mixing.^[^ ^21^, ^22^, ^23^ ^]^ Notably, acoustic mixing has demonstrated advantages in reducing thermal stress and particle damage, enabling uniform processing in minutes rather than hours.^[^ ^20^ ^]^ These earlier implementations laid the physical and conceptual groundwork for the application of RAM in mechanochemistry, where its solvent‐free, scalable nature presents unique opportunities for solid‐state reactivity. Beyond these early applications, RAM has demonstrated exceptional potential for the direct scale‐up of mechanochemical reactions. Successful multi‐gram syntheses have been reported for a variety of targets, including metal–organic frameworks,^[^ ^17^ ^]^ covalent organic frameworks,^[^ ^24^ ^]^ and active pharmaceutical ingredients,^[^ ^25^ ^]^ all achieved without significant changes to reaction protocols. However, its adoption is hindered by unpredictable reaction outcomes. Moreover, real‐time monitoring techniques are essential for understanding reaction mechanisms; however, their application in RAM has been explored only to a limited extent and remains insufficiently developed. Monitoring techniques can be broadly categorized into indirect and direct methods.^[^ ^26^ ^]^ Indirect methods track secondary effects, such as changes in the gas pressure or thermal behavior, while direct methods focus on molecular transformations.^[^ ^27^, ^28^ ^]^ Several powerful analytical tools have been employed for this purpose, including X‐ray Powder Diffraction (PXRD),^[^ ^29^, ^30^, ^31^ ^]^ X‐ray Absorption Spectroscopy (XAS),^[^ ^31^, ^32^ ^]^ Nuclear Magnetic Resonance (NMR),^[^ ^32^, ^33^ ^]^ near‐infrared (NIR),^[^ ^34^ ^]^ and Raman spectroscopy.^[^ ^35^ ^]^ Among these, Raman spectroscopy stands out as a particularly effective in situ technique due to its ability to capture molecular‐level changes with minimal sample preparation.^[^ ^36^ ^]^ High sensitivity to vibrational modes allows for the detection of reaction intermediates,^[^ ^37^, ^38^ ^]^ rapid spectral acquisition enables real‐time reaction tracking,^[^ ^39^, ^40^ ^]^ and nondestructive analysis preserves the sample integrity while providing valuable chemical information.^[^ ^41^, ^42^ ^]^ A recent study demonstrated the integration of in situ Raman spectroscopy with RAM for monitoring a mechanochemical organic reaction, highlighting the adaptation challenges when transferring reactions from ball mills to RAM.^[^ ^43^ ^]^ In particular, the feasibility of in situ Raman spectroscopy in RAM was investigated through the synthesis of α‐aminophosphonates. However, a direct transfer from ball milling was not possible, requiring modifications such as increasing the filling degree, adding liquid‐assisted grinding (LAG)^[^ ^44^, ^45^ ^]^ agents, and adjusting the reaction time. A summary of related studies, including operational parameters such as g‐force, reaction class, and filling degree, is now provided in the Supporting Information (see **10. Literature Comparison**) to contextualize these observations within recent developments. Building on these findings, we systematically explore the critical parameters that determine the viability of in situ Raman monitoring in RAM. Specifically, we seek to identify whether there exists an operational window beyond which certain parameters, such as filling degree, LAG, reaction time, or applied gravitational forces, render the measurement of Raman spectra ineffective. Understanding these limits is essential not only for optimizing reaction conditions and ensuring reliable spectral acquisition but also for establishing RAM as a viable alternative to conventional mechanochemistry. To investigate this operational window, we examined a diverse set of representative reactions, allowing us to assess the robustness and limitations of in situ Raman spectroscopy under varying mechanochemical conditions. This investigation underscores RAM's potential as a versatile synthesis tool across various areas of chemistry.

## 2 Results and Discussion

A key objective of this study was to establish a robust experimental guideline for in situ Raman monitoring of mechanochemical reactions in a Resonant Acoustic Mixer (RAM), by optimizing critical parameters such as vessel material, filling degree, and reaction conditions to ensure reliable spectral acquisition. To fully integrate in situ Raman spectroscopy into RAM, we designed and validated an optimized experimental setup, building upon an already‐established protocol^[^ ^40^ ^]^ for ball milling reactions. To validate its effectiveness, we selected successfully monitored reactions in ball mills, ranging from condensation reactions and material science to catalysis, comparing the molecular vibrational modes to evaluate RAM's capability for mechanochemical reaction monitoring.

### 2.1 Spectra Data Acquisition

Accurate Raman monitoring begins with ex situ spectral characterization of reactants to identify distinct vibrational bands. However, fluorescence from certain compounds can interfere with detection. When fluorescence dominated, reactant substitution or modified conditions improved data quality.

The successful real‐time monitoring of mechanochemical reactions depends on precise control of spectral acquisition parameters. Key factors, including spectral range selection, laser power, number of accumulations, and limited exposure time, must be carefully adjusted to enhance signal clarity. While standard in Raman spectroscopy, techniques such as cosmic ray removal, background noise subtraction, and intensity normalization are particularly crucial for refining spectral data from RAM. Various computational tools and algorithms, such as MATLAB and Origin, enable these corrections, enhancing the reliability of reaction monitoring. Beyond acquisition parameters, rigorous data processing is essential to ensure reproducibility and accuracy.

### 2.2 Development of the Experimental Setup

**Figure 1:**
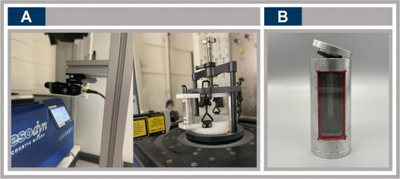
RAM‐Raman experimental setup. A) Raman laser aligned perpendicularly to the sapphire window of the custom vessel within the RAM device, showing vertical and horizontal positioning. B) Custom‐made aluminum vessel with sapphire glass window designed for in situ Raman spectroscopy.

Before the setup construction, the safety aspects of both the RAM system and the Raman laser were assessed. While RAM is generally safer than other mechanochemical methods due to the lack of milling media,^[^ ^46^, ^47^ ^]^ operating it with the door open for laser access introduces risks, including exposure to vibrations and moving parts. The Raman laser also poses hazards from direct eye exposure and beam reflection. To minimize these, all experiments were conducted inside a closed, light‐isolated cabinet. For successful in situ Raman measurements of RAM reactions, the design of specialized tools for the experimental setup is crucial. A key aspect of this setup is the precise alignment of the Raman laser with the RAM device to ensure consistent and accurate signal detection (see Figure 1A). The laser was mounted perpendicularly to the vessel's surface and carefully positioned to ensure optimal signal detection. If placed too high, it would interact only with the upper part of the vessel, missing the reactants. Conversely, if positioned too low, it would strike the bottom of the vessel instead of the reaction mixture. Alongside the spectrometer and RAM, the vessel itself played a critical role in data acquisition. A custom‐made aluminum vessel with a sapphire glass window was employed to facilitate optimal laser penetration and enhance signal detection throughout the mixing process (see Figure 1B).

**Figure 2:**
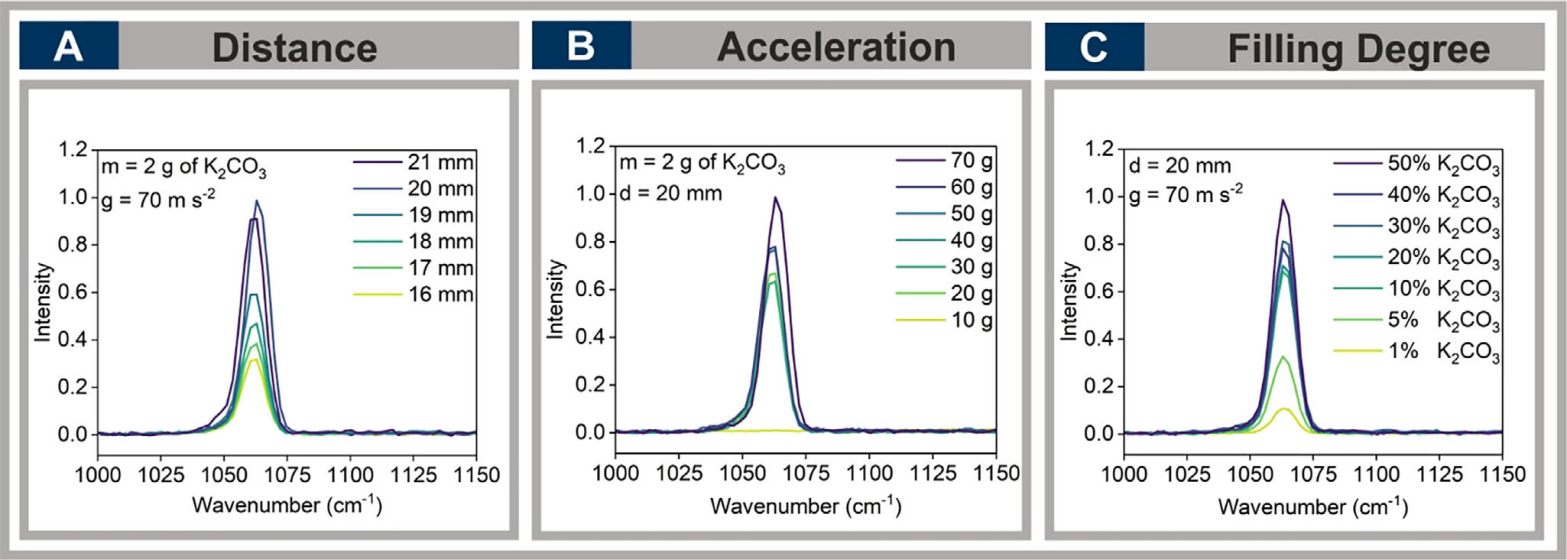
Intensity profiles of K_2_CO_3_ (peak at 1063 cm^−1^) under varying conditions. A) Laser‐to‐vessel distance (16–21 mm); B) g‐forces applied by RAM (10–70 g); C) g‐forces applied by RAM (10–70 g); (C) filling degrees (1–50%).

To ensure accurate in situ measurements, the calibration of laser‐to‐vessel distance was systematically optimized. Proper focusing was essential to achieving an optimal laser spot size, minimizing background noise, and maximizing signal intensity. Potassium carbonate (K_2_CO_3_) was selected due to its distinctive peak at 1063 cm^−1^, allowing precise assessment of signal quality. The laser distance was varied from 16 to 21 mm under constant conditions. Signal intensity gradually increased from 16 mm to 20 mm, reaching its maximum at 20 mm before declining at greater distances (see Figure 2A). Based on these results, a laser distance of 20 mm was determined as optimal for our setup.

### 2.3 Determining the Operational Window for in Situ Raman Spectroscopy with RAM Setup

The first pillar of our operational window is the effect of acceleration, as RAM operates at a constant frequency while g forces vary. Measurements were conducted across a range of 10 to 70 g. The highest signal intensity was observed at 70 g, with forces between 20 and 60 g also producing strong signals without interference, background noise, or signal diminution (see Figure 2B). While measurements outside this range (below 20 g or above 70 g) resulted in slightly weaker intensities or higher background noise, they still provided valuable insights into the system's behavior. By exceeding 80 g, the entire apparatus began to vibrate excessively. This interference was so pronounced that it became impossible to distinguish whether the detected signals originated from molecular vibrations or were simply products of the excessive system movement.

Finally, we investigated the second pillar of our operational window, namely the influence of the filling degree of the vessel on the measurement accuracy. To balance signal strength with minimal reagent consumption and cost efficiency, different filling degrees were tested. A vessel filled to 50% capacity yielded the highest signal intensity. Whereas filling levels between 10% and 40% produced similar intensities, volumes below 10% resulted in weak signals, complicating accurate monitoring (see Figure 2C). Although signals were still detectable at low filling degrees, such as 5%, the decreased intensity limits reliable multicomponent reactions, as minor signals may be overshadowed by noise. Moreover, while higher filling degrees (> 50%) maintain or even improve signal quality, they require greater reagent quantities, increasing material costs significantly.^[^ ^48^ ^]^ Therefore, our focus was on determining the minimum filling degree required to achieve a strong Raman signal. Based on our findings, we establish the threshold at < 10% filling, below which reliable signal detection is no longer feasible.

**Figure 3:**
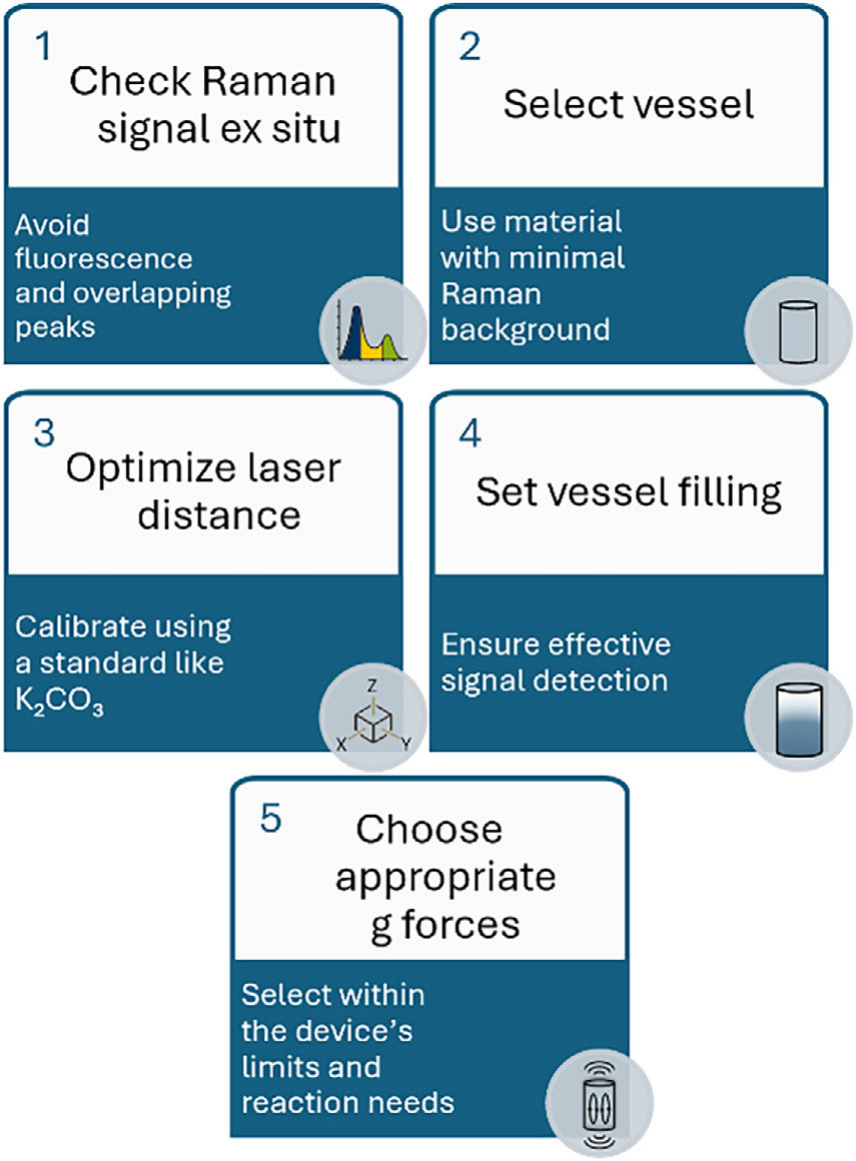
Guideline to measure in situ Raman in RAM.

By optimizing laser positioning (20 mm), g‐force (20–70 g), and filling degree (≥10%), we established a reproducible framework for in situ Raman monitoring using RAM (Figure 3).

### 2.4 Investigation of Representative Reactions

Mechanochemical reactions are well‐established in ball mills, but transferring them to RAM requires adapting key parameters due to fundamental differences in operation. While many reactions have been studied using ball milling devices, these parameters cannot be directly transferred to RAM, as the two systems operate on different principles. Here, we demonstrate the feasibility of adapting RAM for in situ Raman monitoring by examining four distinct examples: organic condensations, chosen for their simple mechanisms, a material synthesis, and a catalytic coupling reaction. To ensure reliable reaction progression and effective monitoring, key parameters were systematically adjusted within the established operational window. This included optimizing the filling degree, selecting appropriate LAG agents, adjusting reaction times, and maintaining acceleration within the defined range, ensuring robust and reproducible Raman signal acquisition.

### 2.5 Knoevenagel Condensation Reaction

To assess whether the selected reactions fall within the established operational window, we investigated the Knoevenagel condensation due to its well‐defined spectroscopic signatures in Raman spectroscopy. The reaction involves the conversion of a carbonyl (C ═ O) functional group into a conjugated C ═ C system, resulting in a characteristic stretching band, corresponding to its simple mechanism and making it suitable for exploration via in situ Raman spectroscopy.^[^ ^49^, ^50^ ^]^ This reaction has been previously studied using ball milling^[^ ^51^ ^]^ and monitored through both in situ Raman spectroscopy and PXRD.

**Figure 4:**
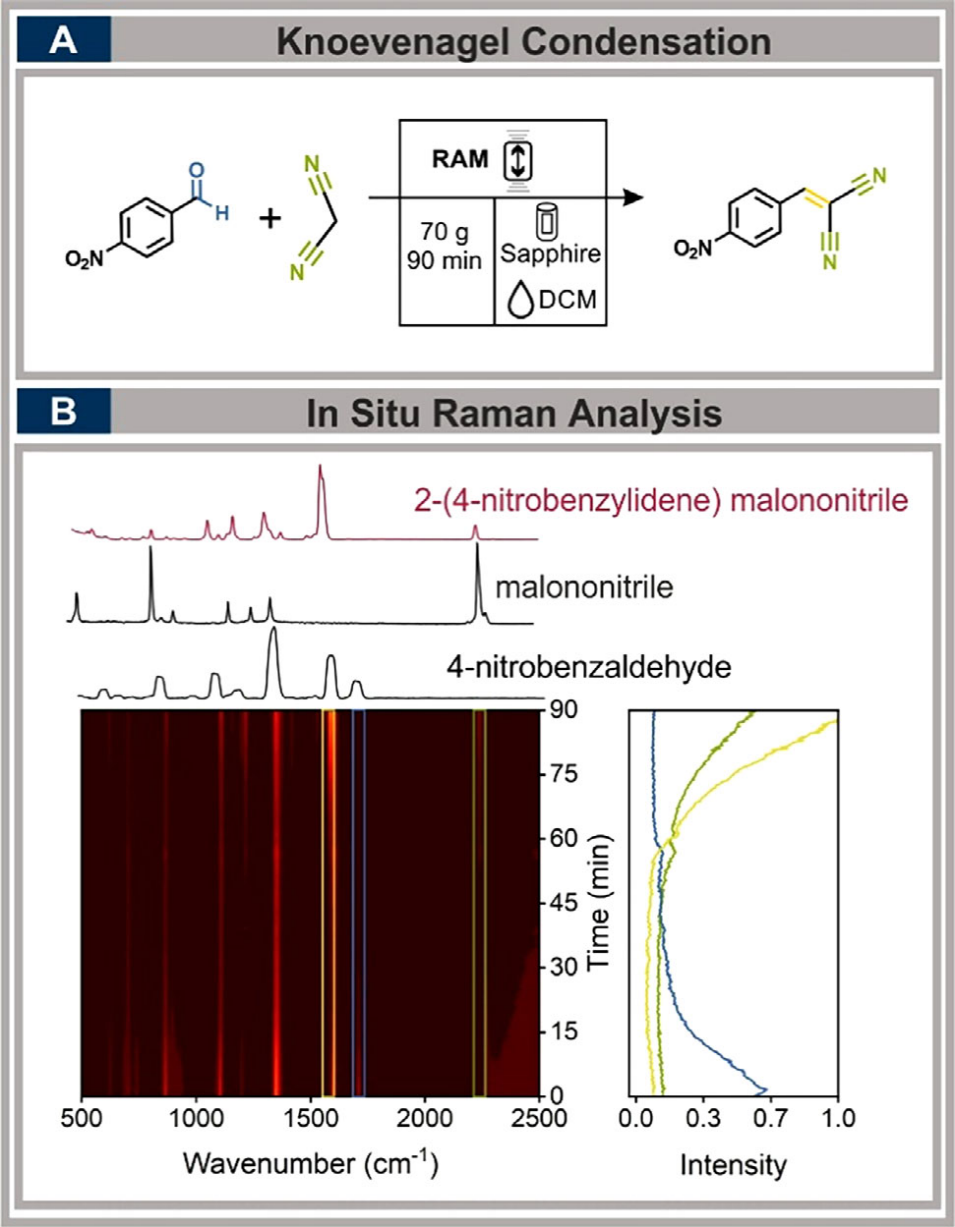
A) Synthesis of (4‐nitrobenzylidene) malononitrile in the RAM. Reaction with 10 mmol of 4‐nitrobenzaldehyde and 10 mmol of malononitrile in the presence of 0.25 µL mg^−1^ DCM as LAG at 70 g for 90 minutes. B) in situ Raman spectra showing carbonyl consumption (1710 cm^−1^, blue), C ═ C bond formation (1577 cm^−1^, yellow), and malononitrile attachment (2238 cm^−1^, green).

We replicated the Knoevenagel condensation of 4‐nitrobenzaldehyde and malononitrile using the RAM‐Raman setup, adapting the reaction to operate within the established operational range. Optimal mixing parameters were determined experimentally, obtaining the best results at 70 g for 90 minutes, with dichloromethane (DCM) serving as LAG (η  =  0.25 µL mg^−1^) (see Figure 4). Additionally, the vessel was filled to 20% of its volume, and subsequently, the reaction was monitored via in situ Raman spectroscopy. The spectroscopic analysis, along with ^1^H‐NMR, provided further confirmation of product formation of 61%. The distinctive consumption of the carbonyl group was observed through the gradual decrease of the peak at 1710 cm^−1^, which decreased to half its initial intensity within approximately 10 minutes and continued decreasing until it disappeared. After around 60 minutes, the formation of the C ═ C bond at 1577 cm^−1^ began to shift, accompanied by the malononitrile attachment at 2238 cm^−1^, corresponding to the second‐order reaction, considered the rate‐determining step.^[^ ^50^, ^51^, ^52^ ^]^

The optimal conditions for the Knoevenagel condensation were found at 70 g, which places it at the upper limit of the operational window, with an extended reaction time of 90 minutes. Likewise, the chosen filling degree of 20% falls within the operation range, positioned toward its lower boundary, while still ensuring strong Raman signal intensity for reliable spectral monitoring. (For detailed results and a quantitative evaluation, refer to the Supporting Information).

### 2.6 Quinoxaline Condensation Reaction

Similarly, mechanochemical condensation reactions have been extensively studied, including quinoxaline formation, which was previously monitored by Friščić and coworkers using a ball mill.^[^ ^53^ ^]^ This reaction involved the condensation of primary amines, specifically benzene−1,2‐diamine, with carbonyl compounds such as benzil, resulting in imine formation.^[^ ^49^ ^]^

**Figure 5:**
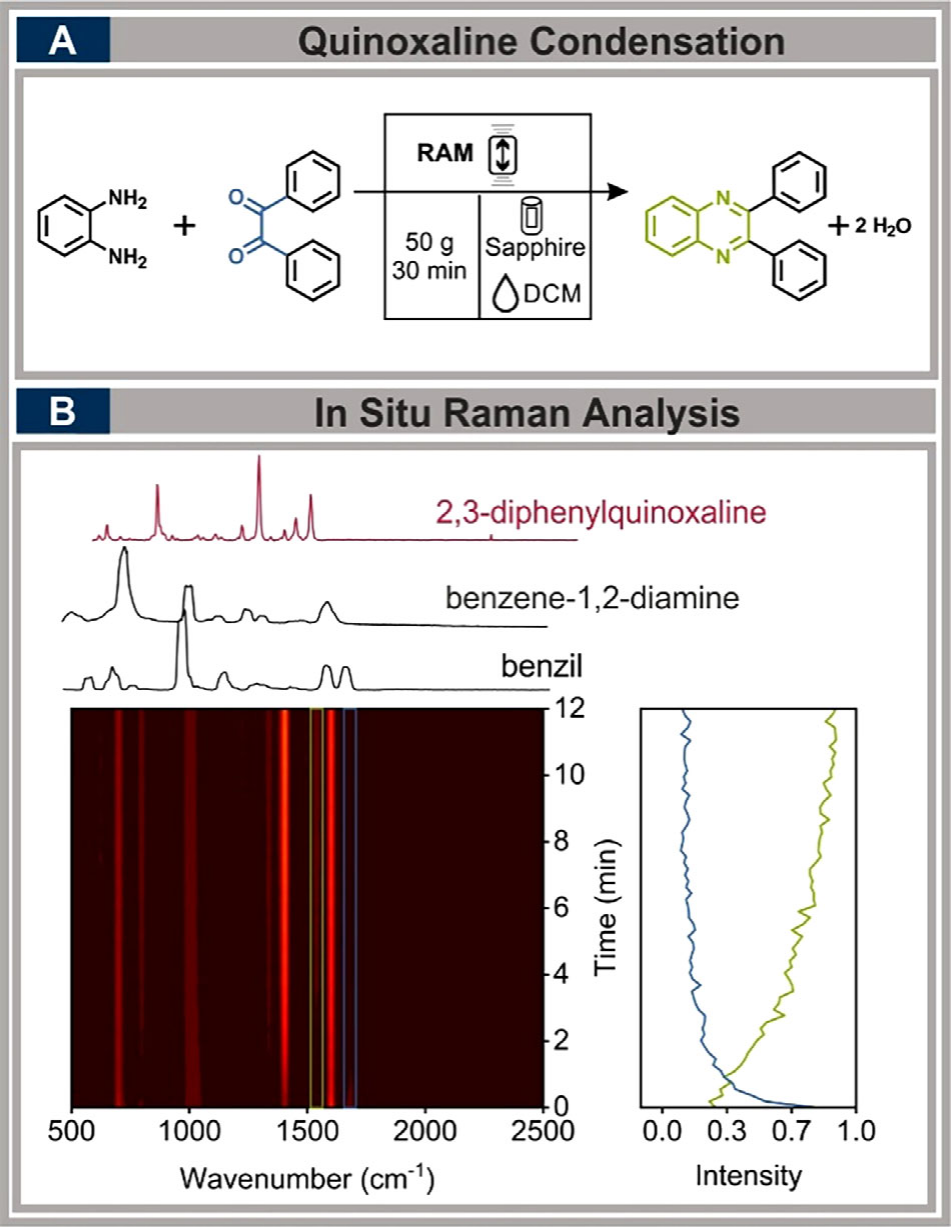
A) Synthesis of 2,3‐diphenylquinoxaline the RAM. Reaction with 5 mmol of benzene−1,2‐diamine and 5 mmol of benzil in the presence of 0.3 µL mg^−1^ DCM as an LAG agent at 50 g for 30 minutes. B) in situ Raman spectra showing quinoxaline formation (1539 cm^−1^, green) and carbonyl consumption (1681 cm^−1^, blue).

In our study, we successfully adapted this reaction to the RAM, achieving optimal conditions at 50 g within 30 minutes and the addition of DCM as LAG agent (η  =  0.30 µL mg^−1^) and a filling degree of 20% (see Figure 5). These optimal conditions are within the operational window, with the g‐forces applied near the lower limit of the range and the volume set close to the minimum required for effective reaction monitoring. Real‐time Raman monitoring of the reaction in the RAM revealed the rapid disappearance of the carbonyl band at 1681 cm^−1^ within the first few minutes. Simultaneously, the formation of quinoxaline was evident from the increasing intensity of the band at 1539 cm^−1^, which increased rapidly during the early stages of mixing and continued to increase until saturation was reached.

Post‐reaction analysis using ^1^H‐NMR with dibromomethane as an internal standard confirmed a product yield of 81%. These results demonstrate that RAM effectively facilitates quinoxaline formation without the need for milling media, preventing contamination, with optimized reaction parameters falling at the lower limit of the operational window. (For detailed results and a quantitative evaluation, refer to the Supporting Information).

### 2.7 Zeolitic Imidazolate Framework (ZIF)

MOFs have gained immense attention due to their exceptional porosity and applications in gas storage, molecular separation, and catalysis.^[^ ^54^ ^]^ Among them, zeolitic imidazolate frameworks (ZIFs) are of particular interest. While mechanochemical synthesis of ZIFs has been extensively studied using ball mills, the potential of RAM for their formation remains largely unexplored.

**Figure 6:**
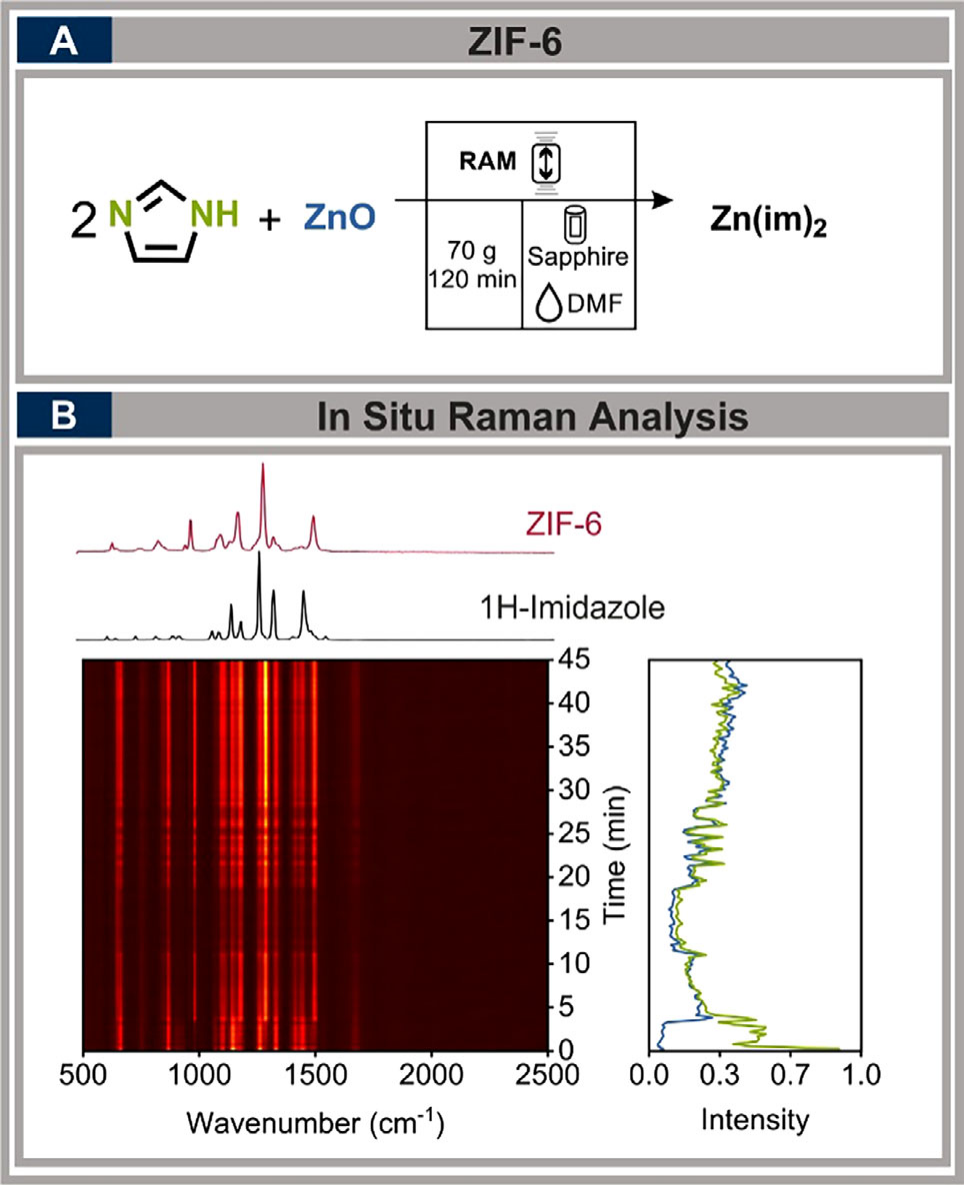
A) Synthesis of ZIF‐6 in the RAM. Reaction with 10 mmol ZnO, 20 mmol imidazole, and 0.1 µL mg^−1^ DMF as LAG at 70 g for 120 minutes; B) Raman spectra showing decreasing intensity at 1146 and 1259 cm^−1^ (green) and increasing intensity at 982, 1181, 1286, and 1497 cm^−1^ (blue).

In this study, we investigated the synthesis of ZIF‐6 under RAM conditions, comparing our findings with previous ball mill studies^[^ ^55^ ^]^ to assess the advantages of this medium‐free method. The reaction involved zinc oxide, imidazole, and dimethylformamide (DMF), with optimum RAM conditions set to 70 g for 120 minutes and a filling degree of 15% to enhance in situ monitoring (see Figure 6). Within just five minutes, significant spectral changes were observed, with bands rapidly increasing and decreasing before stabilizing into a steady progression. The spectral evolution revealed a decrease in intensity for the 1146 cm^−1^ and 1259 cm^−1^ bands, while new bands emerged at 982 cm^−1^, 1181 cm^−1^, 1286 cm^−1^, and 1497 cm^−1^, indicating imidazole coordination with zinc and the development of the framework. Notably, the characteristic spectral features of ZIF‐6 became distinguishable after just 45 minutes, demonstrating the ability of RAM to promote early‐stage transformations. Yields of the isolated final product ranged from 60% to 78%, confirming a consistent and reproducible synthetic route under the tested conditions. (For detailed results and a quantitative evaluation, refer to the Supporting Information.)

### 2.8 Glaser Coupling

The Glaser coupling, a classic oxidative homocoupling reaction of terminal alkynes, is a widely utilized transformation in organic synthesis, particularly for constructing conjugated diynes.^[^ ^56^, ^57^ ^]^ Traditionally, mechanochemical approaches to this reaction have been explored using ball milling, with in situ Raman spectroscopy providing valuable kinetic insights. The Borchard group demonstrated “direct mechanocatalysis”^[^ ^58^ ^]^ by coating milling balls and vessels, which served as a catalyst. This approach facilitated the direct conversion of reactants to products without forming intermediates, enabling in situ monitoring via Raman spectroscopy.^[^ ^56^ ^]^ However, this approach relies on milling media, which influences energy transfer and product formation.

To expand the scope of mechanochemical cross‐coupling reactions and demonstrate the feasibility of in situ Raman monitoring under RAM conditions, we selected the Glaser coupling as a model reaction. Rather than optimizing the yield, our goal was to illustrate that catalytic reactions can be tracked in real‐time using the RAM‐Raman setup.

**Figure 7:**
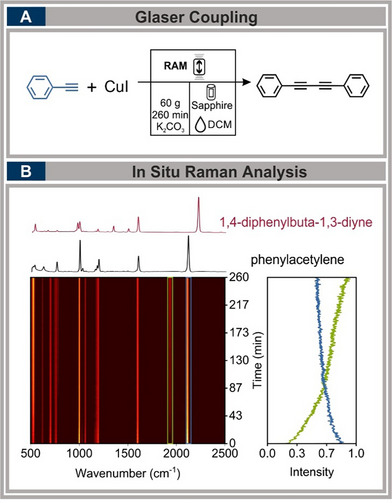
Synthesis of 1,4‐diphenylbuta−1,3‐diyne via Glaser coupling in the RAM. A) Reaction conducted with 20 mmol phenylacetylene, 10% mmol CuI, and 0.20 µL mg^−1^ DCM as LAG for 260 minutes at 60 g; B) Raman spectra showing intermediate formation (1935 cm^−1^, green) and substrate consumption (2114 cm^−1^, blue).

For this study, phenylacetylene, potassium carbonate, and copper(I) iodide were selected as reactants and mixed under RAM conditions. The optimal conditions were achieved at 60 g for 260 minutes, with DCM serving as LAG (η  =  0.20 µL mg^−1^), and a filling degree of 50% (see Figure 7). Real‐time Raman monitoring revealed the formation of an intermediate at 1935 cm^−1^, attributed to the copper‐alkyne complex,^[^ ^49^, ^59^, ^60^ ^]^ which continuously increased without reaching a plateau even after 260 minutes. Simultaneously, a decrease in the intensity of phenylacetylene at 2110 cm^−1^ was observed. Although the final product conversion was modest (12%), the reaction provided valuable insights into intermediate formation and mechanistic behavior under RAM conditions. The limiting factor was the increased filling degree, which, while necessary for effective Raman tracking, significantly reduced the available oxygen inside the vessel. As a result, the copper‐acetylide intermediate could not undergo efficient oxidation, preventing the release of copper and subsequent homocoupling with another phenylacetylene molecule.^[^ ^57^ ^]^ Additionally, the absence of milling media probably altered the energy transfer mechanism, further affecting the reaction progress.

These findings underscore the potential of RAM for monitoring complex catalytic reactions, while also highlighting challenges such as oxygen availability and mechanical energy input. To optimize monitoring, the volume was increased to the maximum level observed in this study, addressing the signal intensity limitation of phenylacetylene. Furthermore, the acceleration applied was near the upper limit of the operational window, facilitating precise monitoring and smooth progression of the reaction. (For detailed results and a quantitative evaluation, refer to the Supporting Information.)

## 3 Conclusion

This study establishes an operational window for in situ Raman spectroscopy in Resonant Acoustic Mixers (RAM), enabling real‐time monitoring of mechanochemical reactions across diverse classes. By optimizing laser‐to‐vessel distance (20 mm), applied g‐forces (20–70 g), and vessel filling degree (≥10%), we developed a robust, reproducible setup. The Knoevenagel and quinoxaline condensations validated these conditions, demonstrating distinct molecular transformations. ZIF‐6 synthesis revealed early‐stage coordination and framework development, while Glaser coupling showcased intermediate detection despite conversion limitations due to oxygen constraints. These findings confirm the feasibility of in situ Raman analysis in the RAM, highlighting its utility for reaction tracking without milling media. The approach reduces contamination risk, supports mechanistic insight, and facilitates solid‐state reactivity under scalable and controlled conditions. By delineating the practical limits for signal acquisition, this work provides a foundation for broader application of RAM in mechanochemistry and paves the way for future advancements in solvent‐free synthesis and real‐time analytical integration.

## Supporting Information

Additional data, figures, and experimental details are available in the Supporting Information document.

## Conflict of Interest

The authors declare no conflicts of interest.

## Supporting information



Supporting Information

## Data Availability

The data that support the findings of this study are available in the supplementary material of this article.
