# Supplementary material for: How to Track Mechanochemical Reactions in Resonant Acoustic Mixers by in Situ Raman Spectroscopy
Source: Chemistry. 2025 Aug 8;31(48):e01336. doi: 10.1002/chem.202501336 (PMC12381690; doi:10.1002/chem.202501336)
Supplement: Supplementary file 1 — Supporting Information [file CHEM-31-e01336-s001.docx]

Supporting Information

How to Track Mechanochemical Reactions in Resonant Acoustic Mixers by *in situ* Raman Spectroscopy

Steffi Krause Hinojosa, Tugce Dogan, Dr. Sven Fabig, Dr. Sven Graetz, and Prof. Dr. Lars Borchardt*

Contents

[1. Materials 2](#_Toc202352449)

[2. Instruments and Methods 2](#_Toc202352450)

[3. Intensity Profiles 5](#_Toc202352451)

[4. Synthetic Procedures 7](#_Toc202352452)

[5. Table of Experiments 9](#_Toc202352453)

[6. Raman Spectra 11](#_Toc202352454)

[7. NMR Spectra 19](#_Toc202352455)

[8. GC-MS 21](#_Toc202352456)

[9. SEM and EDS 22](#_Toc202352457)

[10. Literature Comparison 24](#_Toc202352458)

[11. Supporting References 25](#_Toc202352459)

# Materials

The chemicals and reagents were sourced from commercial suppliers and used without further modification: Benzene‑1,2‑diamine (TCI, 98 % purity), benzil (abcr, 98 % purity), CuI (Fluorochem, 98 % purity), 1H-imidazole (Acros Organic, 99 % purity), iron(III) chloride (Alfa Aesar, 99 % purity), malononitrile (Thermo Scientific, 99 % purity), 4‑nitrobenzaldehyde (Sigma Aldrich, 98 % purity), phenylacetylene (Aldrich, 98 % purity), potassium carbonate (Fischer Chemical, 99.5 % purity), zinc oxide (Alfa Aesar 98 % purity). These materials served as the basis for the experiments outlined in this work.

# Instruments and Methods

The Resonant Acoustic Mixer (RAM) was purchased from ResodynAcoustic® Mixers as a LabRAM II model. Its performance oscillates between 10 g and 100 g of acceleration, with a frequency of approximately 60 Hz.

Raman spectra were obtained using a RENISHAW inVia Qontor Raman. For the *ex situ* analysis, the objective used was 50x with a wavelength of 785 nm. The power of the laser was set between 5 % and 50 % based on the sample, exposing the sample for 10 seconds and 5 accumulations per sample.

For the *in situ* measurements, the utilized laser was connected to a fiber optic. Also, the wavelength was 785 nm with a laser power of 100 %. The exposure time for the reaction was 1 second with a 10 to 15 accumulations per round. The data processing after the Raman measurements, including cosmic ray removal and data acquisition performed using WiRe 5.1 software, followed by smoothing and baseline correction using MATLAB 9.9.

Nuclear magnetic resonance (NMR) spectroscopy, including ^1^H‑NMR and ^13^C‑NMR spectra, was utilized for analysis. The ^1^H and ^13^C NMR spectra were obtained using a Bruker Avance III HD spectrometer operating at 400 MHz, with CDCl_3_ as the solvent. Also, the ^1^H and ^13^C spectra were internally calibrated to the residual solvent peak and referenced to dibromomethane (CH_2_Br_2_). Chemical shifts are reported in parts per million (ppm) relative to CH_2_Br_2_. The spectral data is presented as: s = singlet, d = doublet, dd = doublet of doublets, m = multiplet, with coupling constants reported in Hertz (Hz).

Gas chromatograph‑mass spectrometry (GC‑MS) analysis was conducted using a Shimadzu NEXIS 2030 gas chromatograph equipped with a 30 m 0.25 mm ID, 5 % diphenyl and 95 % dimethyl polysiloxane stationary phase. Electron ionization (EI) was employed with an injector temperature maintained at 200 °C. For the measurements, 1 mg of crude sample was dissolved in 1 mL of dichloromethane (DCM), filtered through cotton, and transferred into a gas chromatography vial for analysis.

High‑performance liquid chromatography (HPLC) was performed using a Shimadzu Nexera LC‑40 lite system. The stationary phase consisted of a Nucleodur C18 reversed‑phase column (3 µm particle size) from Macherey‑Nagel. The mobile phase was an isocratic mixture of 85 % acetonitrile and 15 %, with a flow rate of 1 mL/min. For analysis, 1 mg of the crude product was dissolved in a 65:35 acetonitrile/water mixture, acidified with 0.1 % trifluoroacetic acid (TFA), filtered through a syringe filter, and transferred to an HPLC vial.

Scanning electron microscopy (SEM) images were obtained at an accelerating voltage of 15 kV using a high‑resolution JOEL JSM‑IT800SHL scanning electron microscope. The energy-dispersive X-ray spectra (EDX) were executed by the Oxford Ultim Max with a silicon drift detector at a working distance (WD) of 10 mm.

Energy‑dispersive X‑ray spectroscopy (EDS) was performed using an Oxford Ultim Max Silicon Drift Detector (SDD) as the primary detector.

# Intensity Profiles


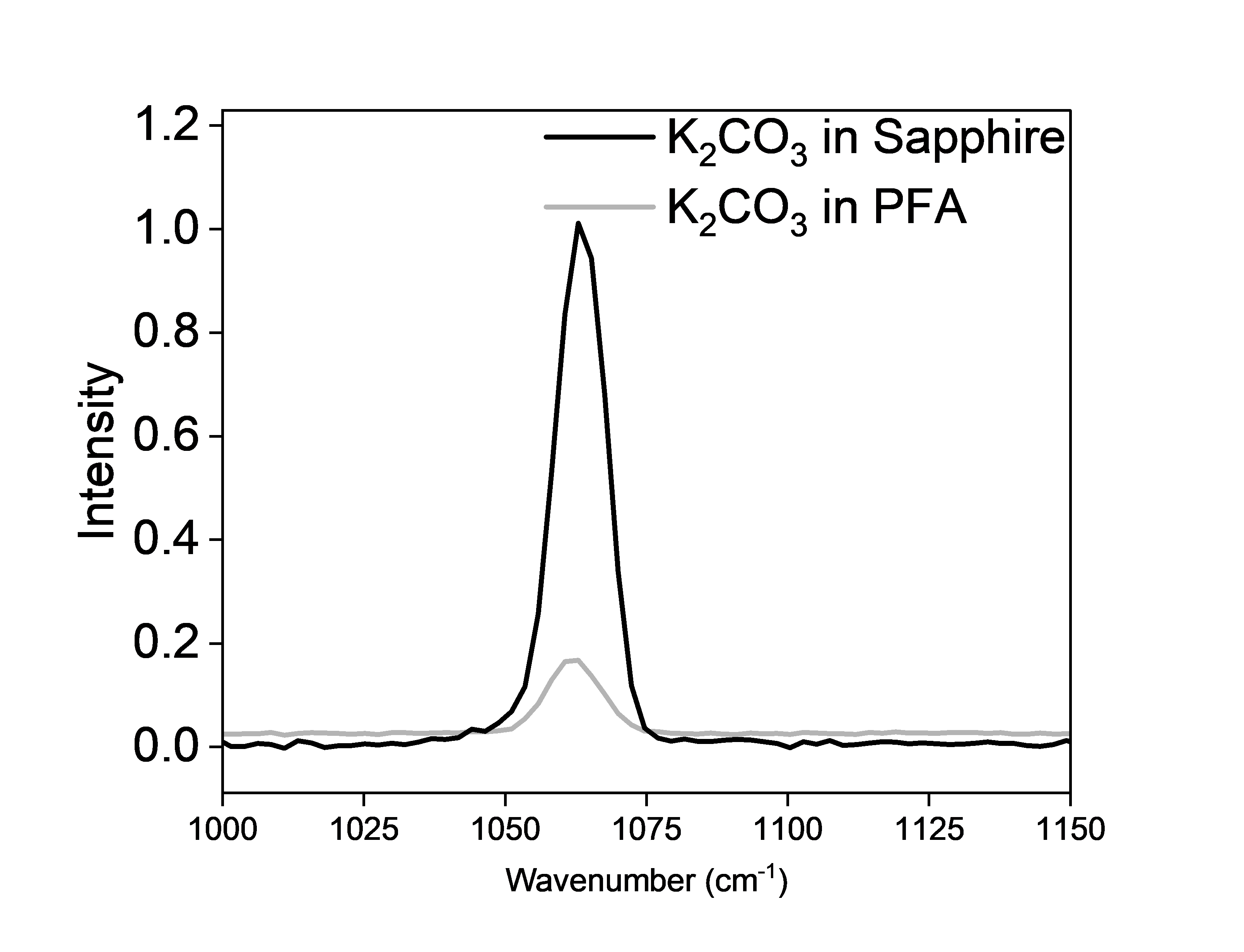


Figure S1: Intensity profile obtained from measurements of K_2_CO_3_, showing a signal at 1063 cm^-1^ and comparing its intensity using different vessel materials: PFA and the custom-made aluminum vessel with a sapphire glass window.


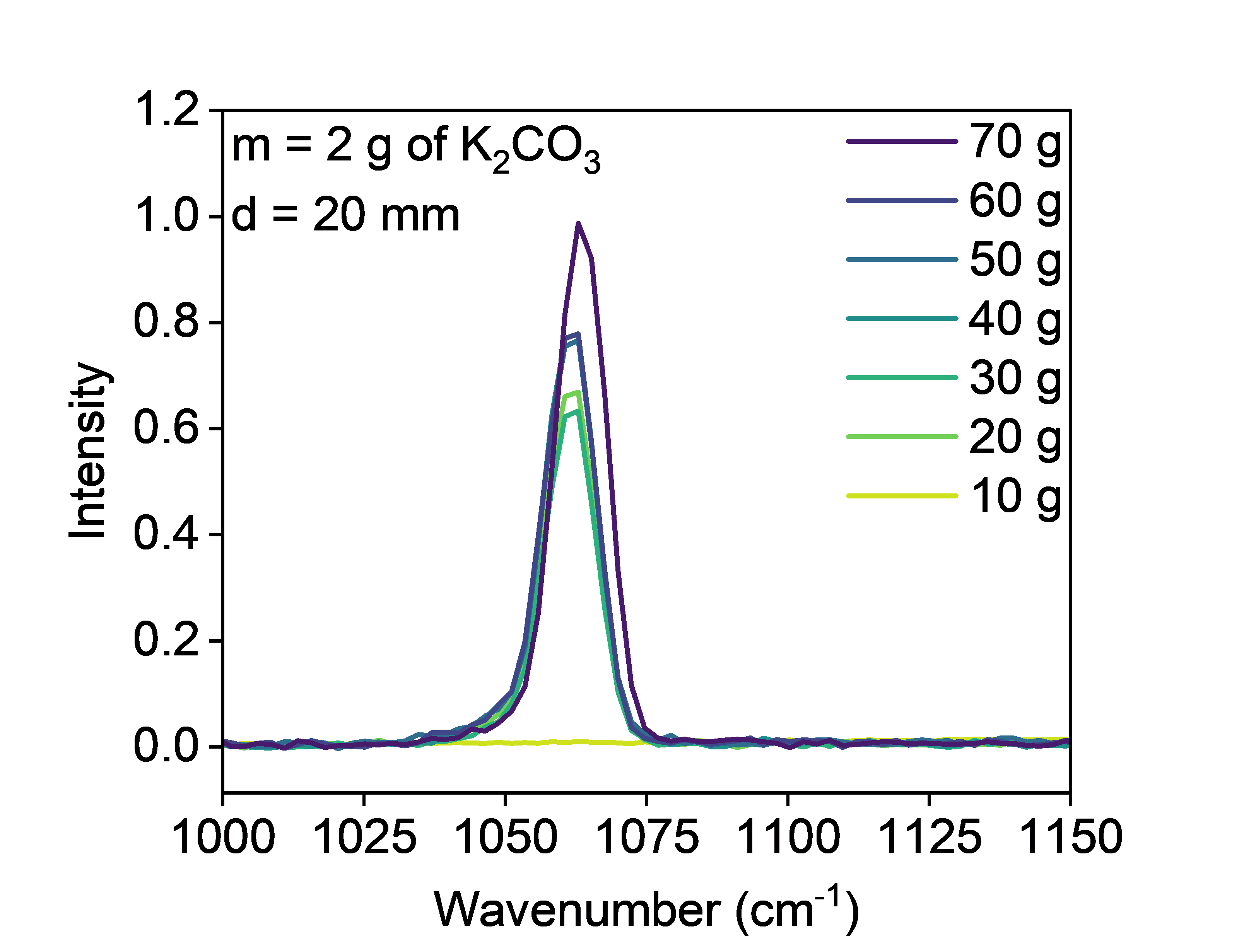


Figure S2: Intensity profile obtained from measurements of K_2_CO_3_, showing a signal at 1063 cm^-1^ and the effect of different g forces applied by the RAM, from 10 g to 70 g, with intensities above this range being unmeasurable.


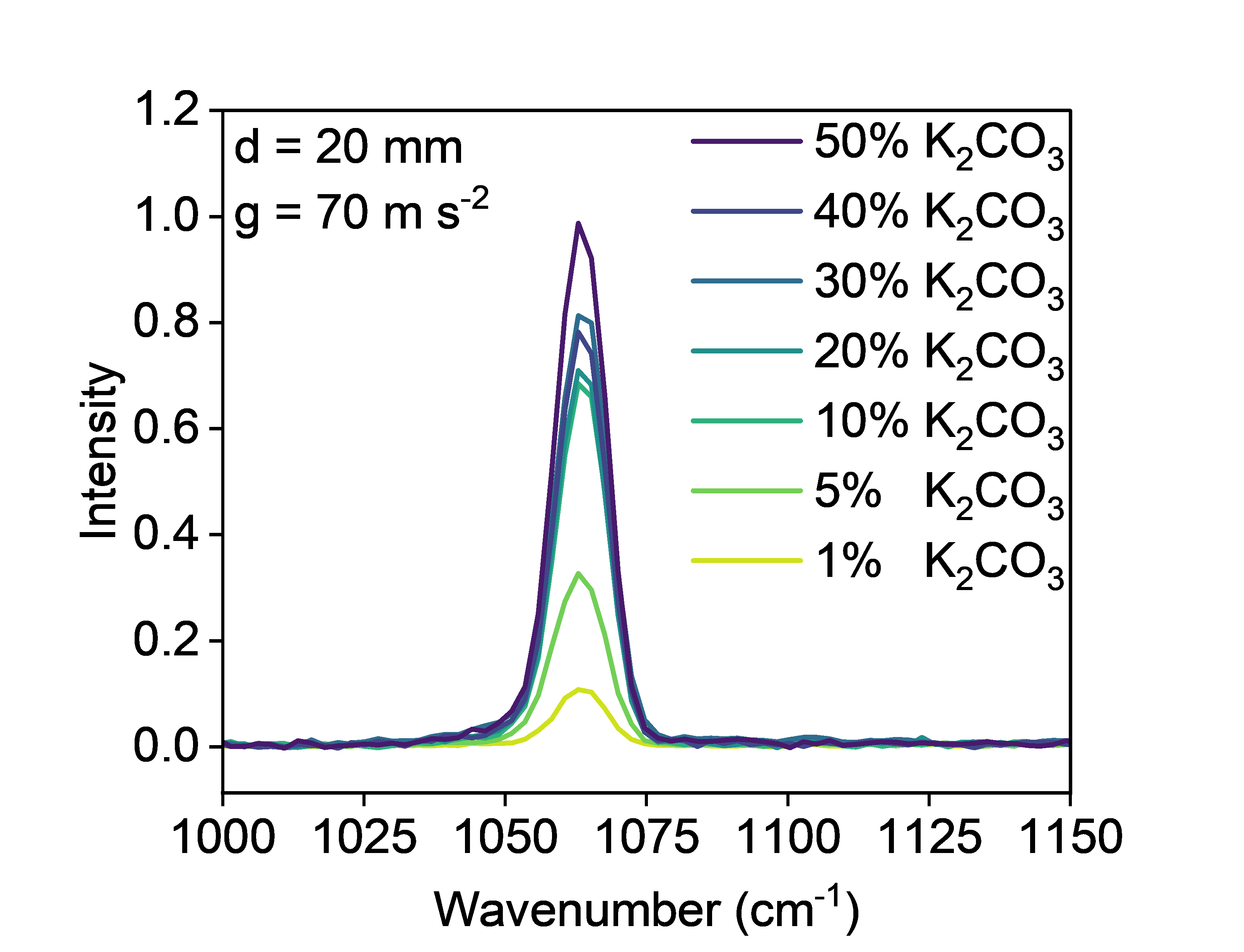


Figure S3: Intensity profile obtained from measurements of K_2_CO_3_, showing a signal at 1063 cm^-1^ and the effect of K_2_CO_3_ filling degree, varying from 1 % to 50 %.


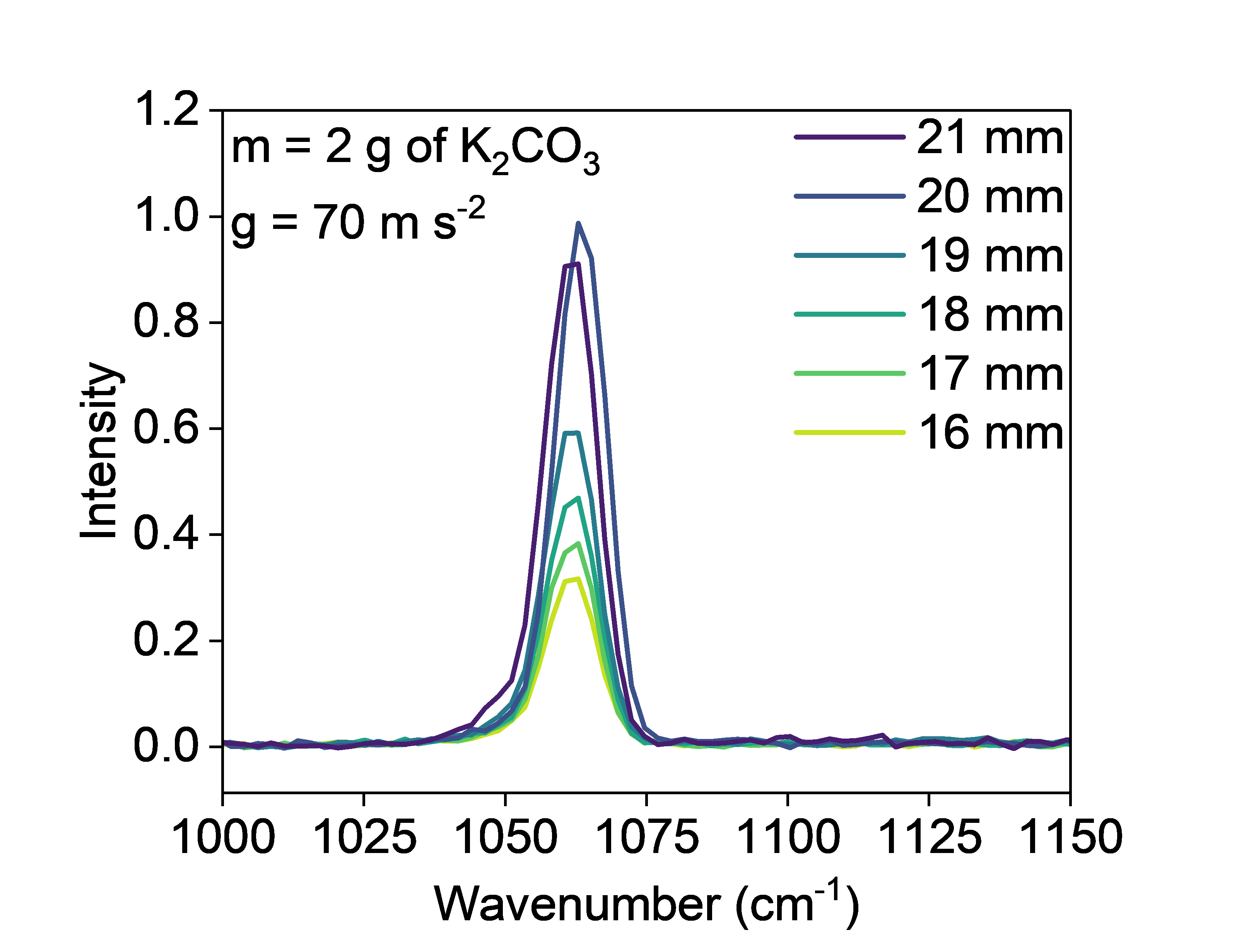


Figure S4: Intensity profile obtained from measurements of K_2_CO_3_, showing a signal at 1063 cm^-1^ and variation in the laser-to-vessel distance, ranging from 16 mm to 21 mm.

# Synthetic Procedures

*2-(4-Nitrobenzylidene) malononitrile*

The synthesis of 2‑(4‑nitrobenyzlidene) malononitrile was carried out by mixing 4‑nitrobenzaldehyde (1.60 g, 10.58 mmol, 1 eq.) and malononitrile (698.95 mg, 10.58 mmol, 1 eq.) in presence of DCM as LAG agent (575 µL, η = 0.25 µL/mg) in an aluminum vessel with sapphire glass at 70 g for 90 min in the RAM. The reaction progress was monitored *in situ* using a Raman spectrometer. At the end of the reaction, the product was carefully scraped from the vessel and left to dry at room temperature for several minutes until fully desiccated. The reaction yielded an orange‑yellow solid (2160.9 g, 9.8 mmol, 61.0 % yield). In addition to the Raman spectrum, the final product was confirmed using GC‑MS. ^1^H‑NMR (400 MHz, CDCl_3_): δ 8.39 (d, J = 8.6, 2H), 8.07 (d, J = 8.8, 2H), 7.88 (s, 1H). ^13^C‑NMR (101 MHz, CDCl_3_): δ 157.0, 150.5, 135.9, 131.5, 124.8, 112.8, 111.7, 87.7.

*2,3‑Diphenylquinoxaline*

The synthesis of 2,3-diphenylquinoxaline was carried out by mixing benzene-1,2-diamine (540.72 mg, 5.00 mmol, 1 eq.) and benzil (1.05 g, 5.00 mmol, 1 eq.) in presence of dichloromethane (DCM) as LAG agent (160 µL, η = 0.1 µL/mg) in an aluminum vessel with sapphire glass at 50 g for 30 min in the RAM. The reaction progress was monitored *in situ* using a Raman spectrometer. At the end of the reaction, the product was carefully scraped from the vessel and left to dry at room temperature for several minutes until fully desiccated. The reaction yielded a light-yellow powder (1325.5 mg, 4.7 mmol, 81 % yield). ^1^H‑NMR (400 MHz, CDCl_3_): δ 8.10 (dt, J = 6.4, 3.2 Hz, 1H), 7.78 (dq, J = 6.4, 3.0 Hz, 1H), 7.56 – 7.47 (m, 2H), 7.38 – 7.29 (m, 3H). ^13^C‑NMR (75 MHz, CDCl_3_): δ 153.6, 141.4, 139.2, 130.0, 129.4, 128.9, 128.4.

*Zeolitic imidazolate framework (ZIF‑6)*

The synthesis of ZIF-6 was carried out by mixing zinc oxide (813.79 mg, 10.00 mmol, 1 eq.) and 1H‑imidazole (1.36 g, 20.00 mmol, 2 eq.) in presence of dimethylformamide (DMF) as LAG agent (230 µL, η = 0.1 µL/mg) in an aluminum vessel with sapphire glass at 70 g for 120 min in the RAM. The reaction progress was monitored *in situ* using a Raman spectrometer. At the end of the reaction, the product was carefully scraped from the vessel and left to dry at room temperature for several minutes until fully desiccated. The reaction yielded a white powder (2469.2 mg, 12.4 mmol, 73.6 % yield). In addition to the Raman spectrum, the final product was characterized using PXRD and compared with the pattern from the Cambridge Structural Database (CSD), with Database identifier EQOCOC, as well as the characterization with SEM.

*1,4‑Diphenylbuta‑1,3‑diyne*

The synthesis of 1,4‑diphenylbuta‑1,3‑diyne was carried out by mixing phenylacetylene (2.04 g, 20.00 mmol, 1 eq.), copper(I) iodide (380.90 mg, 2.00 mmol, 0.1 eq.), and potassium carbonate (2.76 g, 20.00 mmol, 1 eq.) in presence of dichloromethane (DCM) as LAG agent (1036 µL, η = 0.2 µL/mg) in an aluminum vessel with sapphire glass at 60 g for 260 min in the RAM. The reaction progress was monitored *in situ* using a Raman spectrometer. At the end of the reaction, the product was washed with DCM and concentrated under reduced pressure. The reaction yielded a light-yellow solid crystal. In addition to the Raman spectrum, the final product was confirmed and characterized by HPLC.

# Table of Experiments

Table S1: Overview of the obtained mass and yield for the synthesis of (4-nitrobenzylidene) malononitrile.

Unless otherwise stated, the reaction was carried out in the RAM using a custom-made aluminum vessel with a sapphire glass window. The substrate amount was set at 10.58 mmol with one equivalent of each substrate, and LAG was performed with η = 0.25 µL mg^-1^. The final product and the yields were confirmed using GC-MS.

| **Sample** | **Time (min)** | **Forces** | **Substrate 1** | **Mass (mg)** | **Substrate 2** | **Mass (mg)** | **LAG** | **Volume (µL)** | **Mass obtained (mg)** | |
| --- | --- | --- | --- | --- | --- | --- | --- | --- | --- | --- |
| KC-01 | 90 | 70 g | Malononitrile | 698 | 4-NBA | 1597 | DCM | 575 | 2107.0 |  |
| KC-02 | 90 | 70 g | Malononitrile | 696 | 4-NBA | 1599 | DCM | 575 | 1948.1 |  |

4-NBA = 4-Nitrobenzaldehyde; DCM = Dichloromethane

Table S2:Overview of the obtained mass and yield for the synthesis of 2,3-diphenylquinoxaline.

Unless otherwise stated, the reaction was carried out in the RAM using a custom-made aluminum vessel with a sapphire glass window. The substrate amount was set at 5 mmol, and LAG was performed with η = 0.3 µL mg^-1^. The final product and the yields were confirmed and calculated using ^1^H-NMR spectra, with dibromomethane as the internal standard.

| **Sample** | **Time (min)** | **Forces** | **Substrate 1** | **Mass (mg)** | **Substrate 2** | **Mass (mg)** | **LAG** | **Volume (µL)** | **Mass obtained (mg)** | **Yield (%)** |
| --- | --- | --- | --- | --- | --- | --- | --- | --- | --- | --- |
| QR-01 | 20 | 50 g | BDA | 541.0 | Benzil | 1053.0 | DCM | 477.0 | 1435.5 | 67.0 |
| QR-02 | 30 | 50 g | BDA | 553.0 | Benzil | 1052.0 | DCM | 477.0 | 1325.5 | 81.0 |

BDA = Benzene-1,2‑diamine; DCM = Dichloromethane

Table S3: Overview of the obtained mass and yield for the synthesis of zeolitic imidazolate framework (ZIF-6). Unless otherwise stated, the reaction was carried out in the RAM using a custom-made aluminum vessel with a sapphire glass window. The substrate amount was set at 10 mmol with one equivalent of each substrate, and LAG was performed with η = 0.1 µL mg^-1^. The final product was confirmed by PXRD, and the yields were determined through gravimetric analysis.

| **Sample** | **Time (min)** | **Forces** | **Substrate 1** | **Mass (mg)** | **Substrate 2** | **Mass (mg)** | **LAG** | **Volume (µL)** | **Mass obtained (mg)** | **Yield (%)** |
| --- | --- | --- | --- | --- | --- | --- | --- | --- | --- | --- |
| ZIF-01 | 120 | 60 g | Zinc oxide | 813 | Imidazole | 1357 | DMF | 652.0 | 2603.1 | 77.2 |
| ZIF-02 | 120 | 60 g | Zinc oxide | 814 | Imidazole | 1368 | DMF | 652.0 | 1982.4 | 59.0 |

DMF = Dimethyl formamide, ^[a]^ η = 0.4 µL/mg

Table S4: Overview of the obtained mass and yield for the synthesis of 1,4-diphenyl-1,3-diyne. Unless otherwise stated, the reaction was carried out in the RAM for 260 minutes at 60 g using a custom-made aluminum vessel with a sapphire glass window. The substrate amount was set at 20 mmol with η = 0.3 µL mg^-1^ LAG. The final product was confirmed by GC-MS, and the yields were determined through HPLC.

| **Sample** | **Substrate 1** | **Mass (mg)** | **Substrate 2** | **Mass (mg)** | **Substrate 3** | **Mass (g)** | **LAG** | **Volume (µL)** | **Mass obtained (mg)** |
| --- | --- | --- | --- | --- | --- | --- | --- | --- | --- |
| GC-03 | PA | 510.7^[a]^ | CuI | 191.0 | K_2_CO_3_ | 2.0 | DCM | 540 | 55.8 |
| GC-04 | PA | 2040.0 | CuI | 388.9 | K_2_CO_3_ | 2.8 | DCM | 1200 | 7.3 |

PA = Phenylacetylene, DCM = Dichloromethane, ^[a]^ 5 mmol

# Raman Spectra

All raw data are included in a cloud‑based repository at the following link: https://github.com/Ste-KH/RAM-Raman_RawData

- 1. **Knoevenagel Condensation**


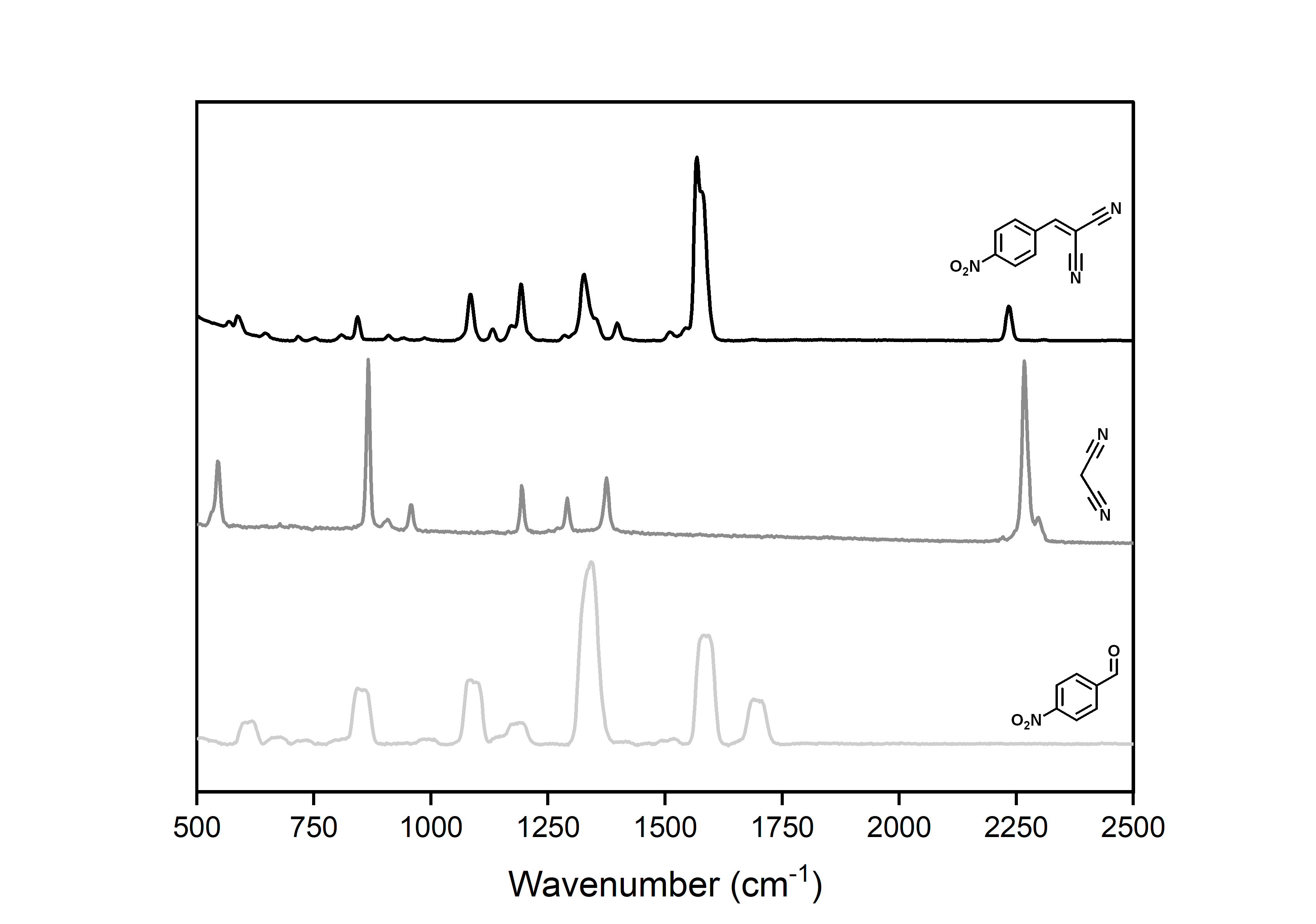


**Figure S5**: Ex situ spectrum of the Knoevenagel condensation. The spectrum of synthesized 2-(4‑nitrobenzylidene) malononitrile is shown at the top, followed by that of malononitrile, and then the spectrum of 4‑nitrobenzaldehyde presented at the bottom.


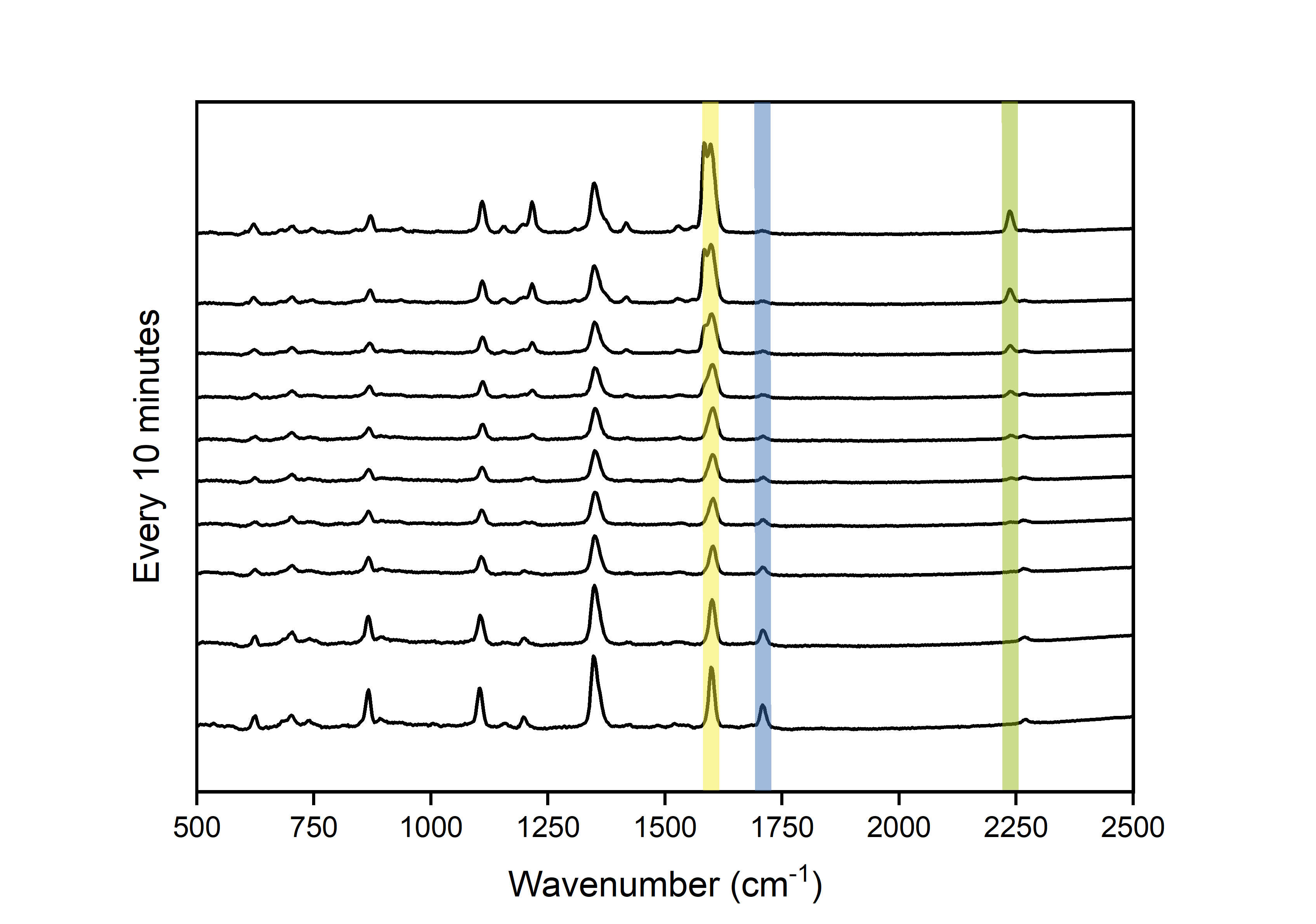


**Figure S6**: Time-resolved Raman spectra measured during the synthesis of 2-(4-nitrobenzylidene) malononitrile.


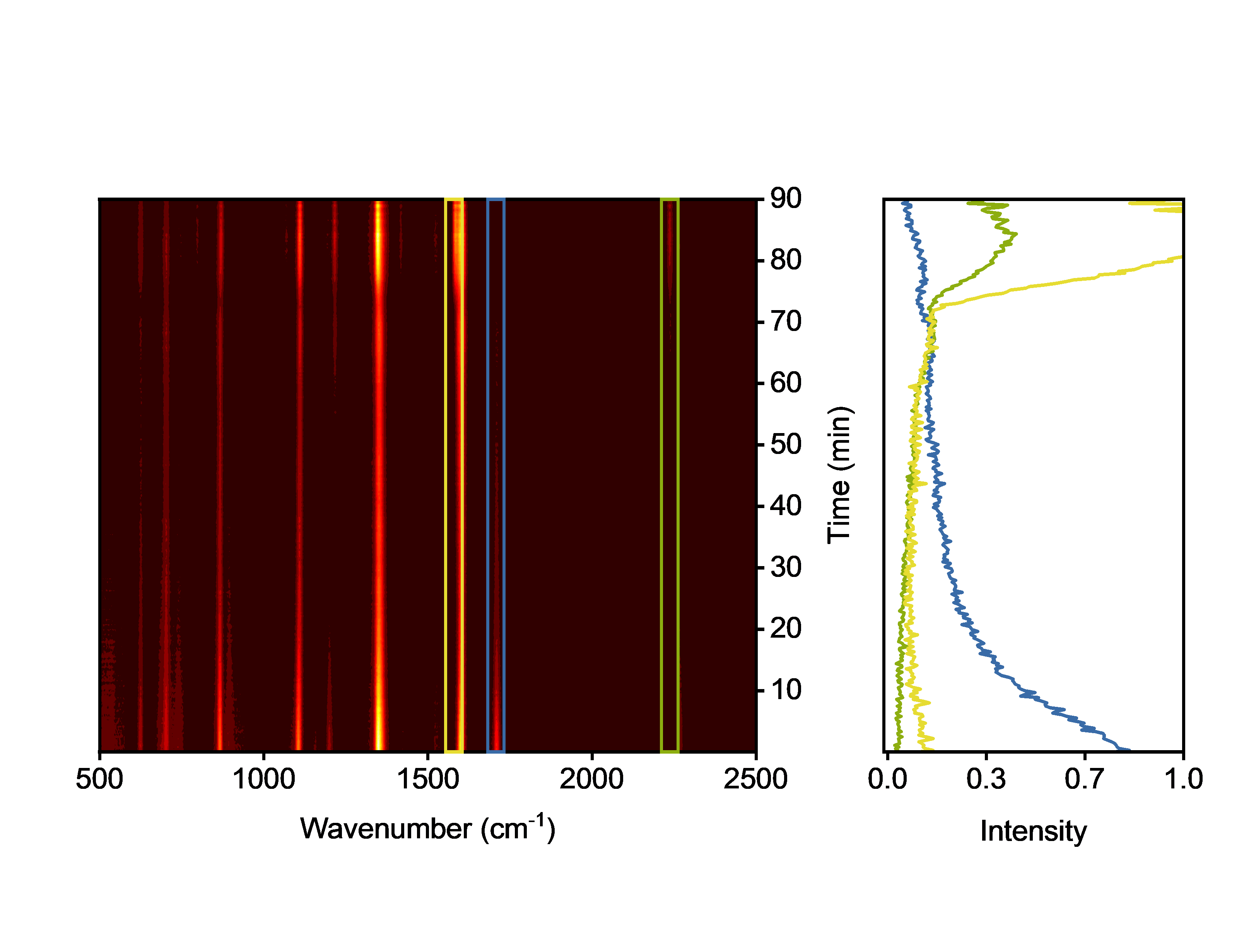


Figure S7: Duplicated synthesis of (4-nitrobenzylidene) malononitrile under mechanochemical conditions in the RAM. The same conditions were used to replicate the reaction. This graph presents the full *in situ* Raman spectrum, depicting the decreasing intensity of the carbonyl group at 1710 cm^‑1^ (blue), as well as the increasing formation of the C=C bond at 1577 cm^‑1^ (yellow), and the attachment of the malononitrile at 2238 cm^‑1^ (green).

1. **Quinoxaline Condensation**


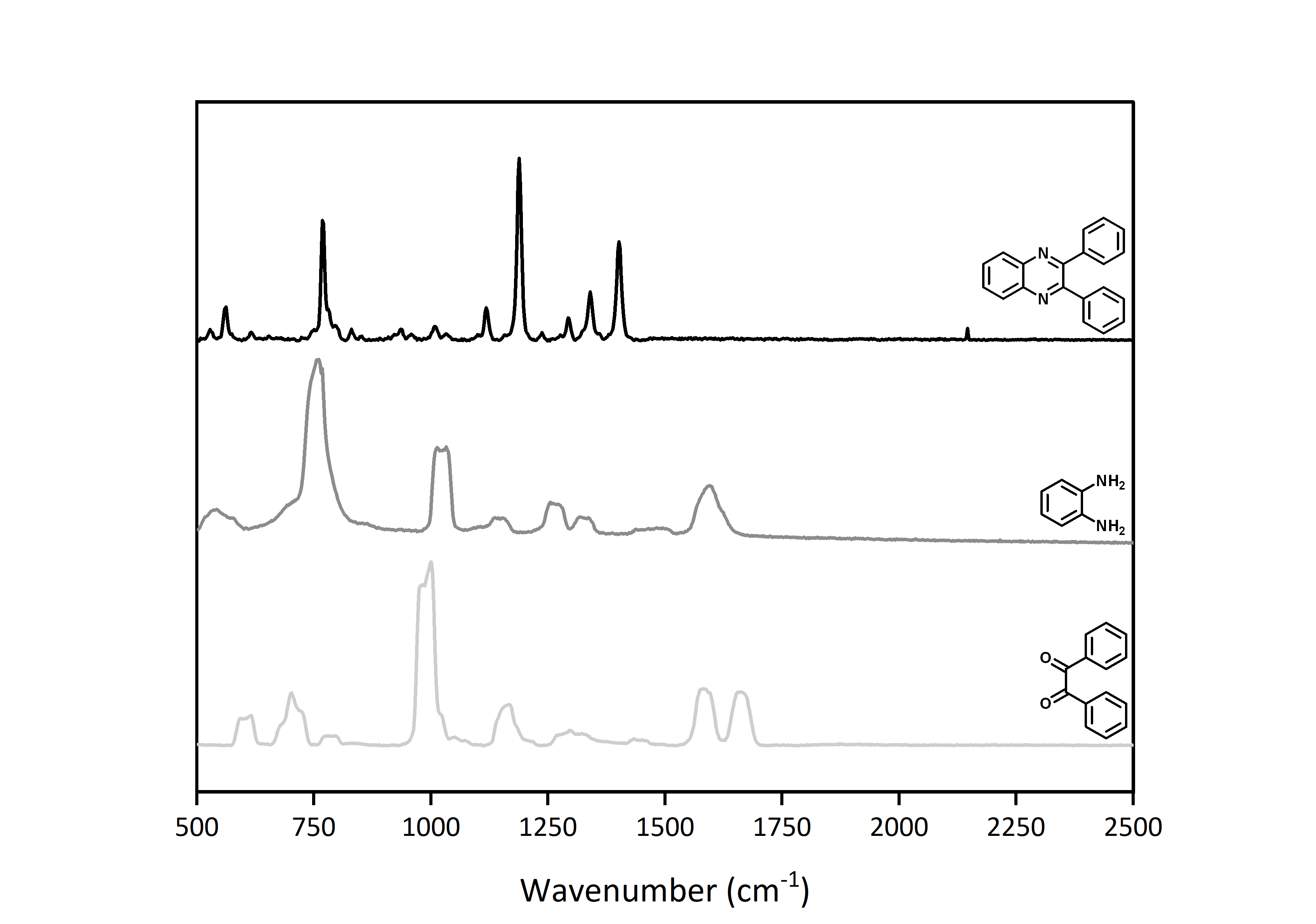


**Figure S8**: Ex situ spectrum of the reaction of the quinoxaline condensation. The spectrum of synthesized 2,3‑diphenylquinoxaline is shown at the top, followed by that of benzene-1,2-diamine, and then the spectrum of benzil presented at the bottom.


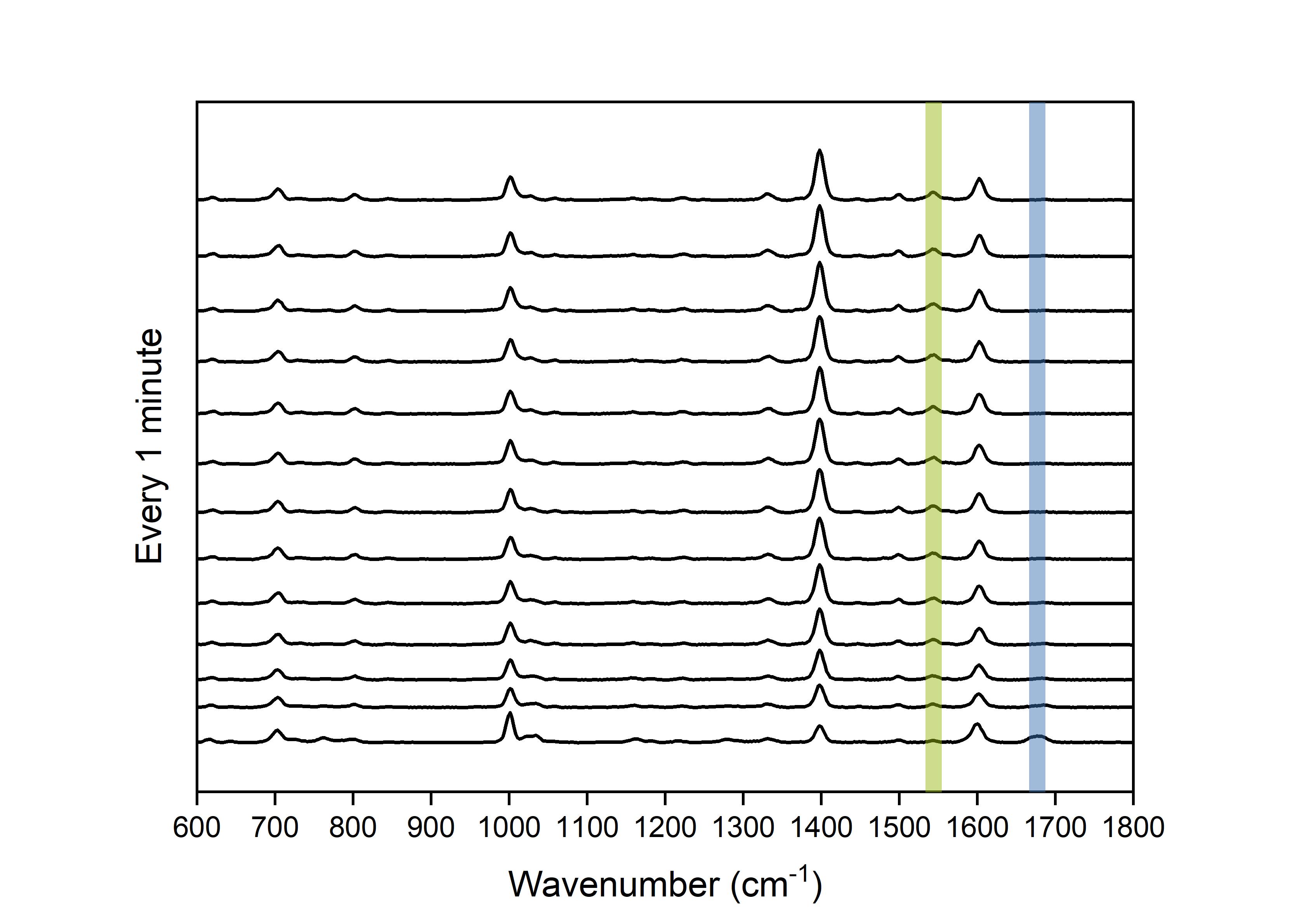


**Figure S9**: Time-resolved Raman spectra measured during the quinoxaline condensation,

resulting in the formation of 2,3‑diphenylquinoxaline.


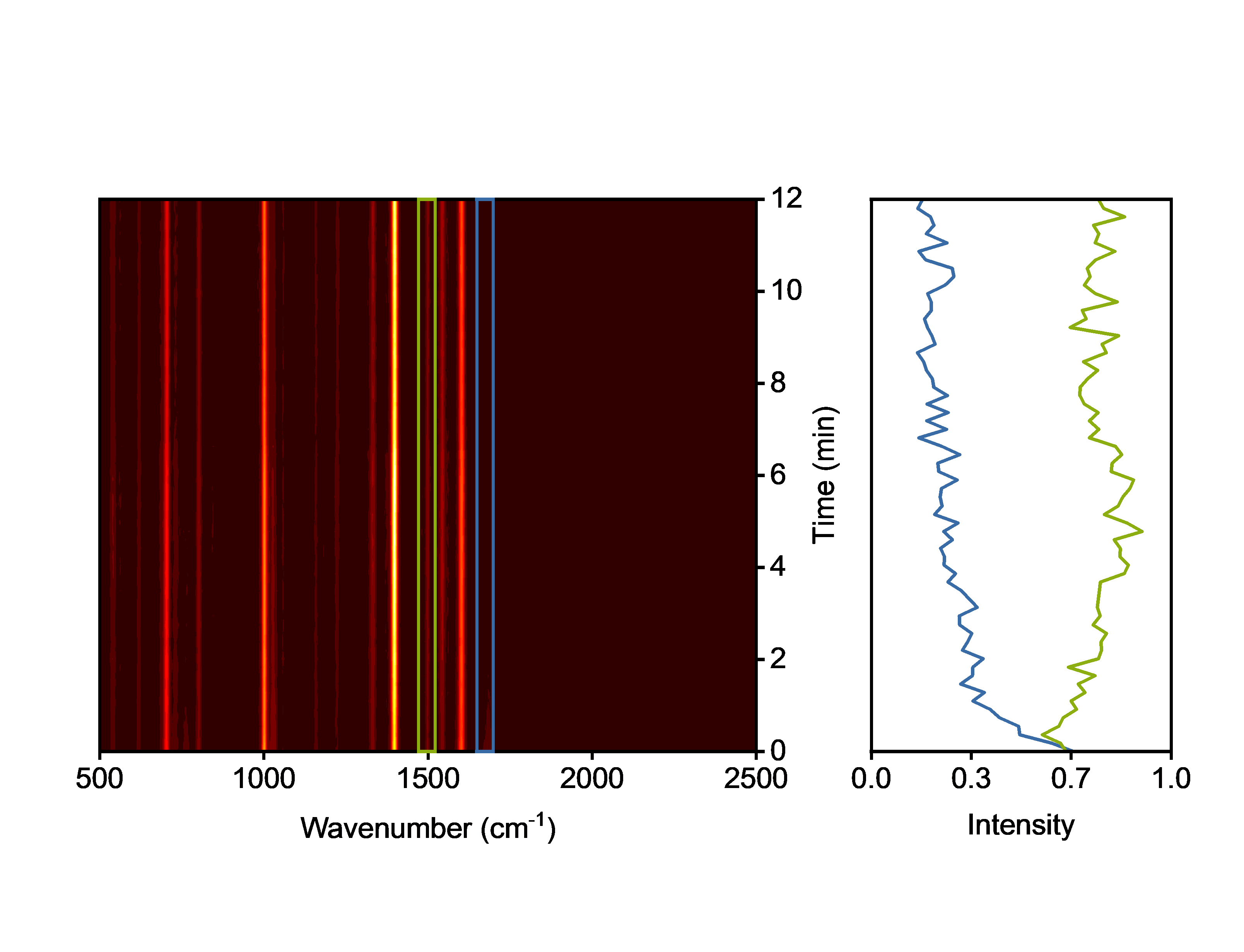


Figure S10: Duplicated synthesis of 2,3‑diphenylquinoxaline under mechanochemical conditions in the RAM. The same conditions were used to replicate the reaction. This graph presents the full in situ Raman spectrum, highlighting at 1539 cm^‑1^ (green) the increasing formation of quinoxaline and at 1681 cm^‑1^ (blue) the decreasing intensity of the carbonyl group over time.

1. **MOF: ZIF-6**


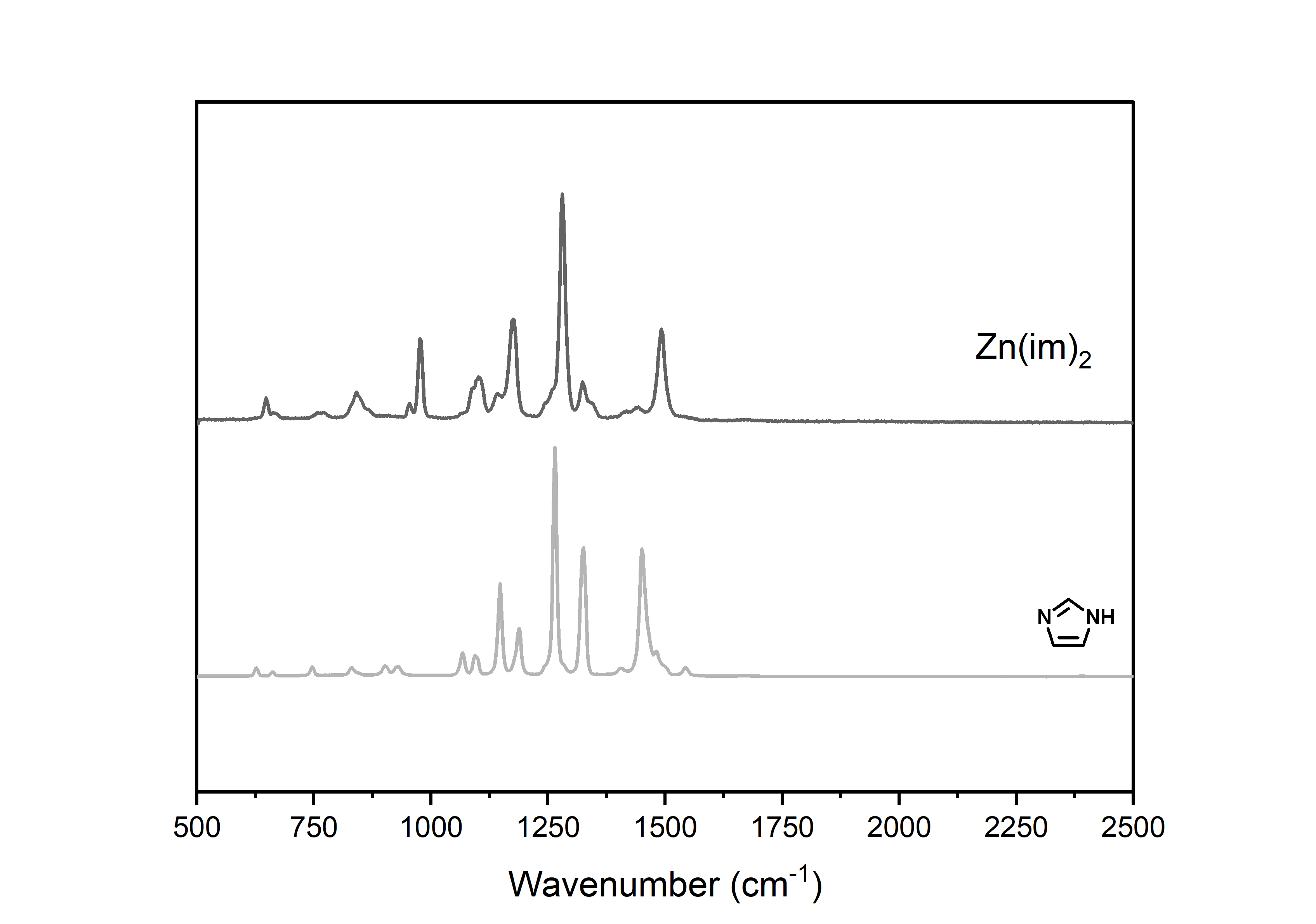


**Figure S11**: Ex situ spectrum of the synthesis of ZIF-6.

The spectrum of ZIF‑6 is shown at the top, followed by that of zinc oxide, and then the spectrum of 1H‑imidazol presented at the bottom.


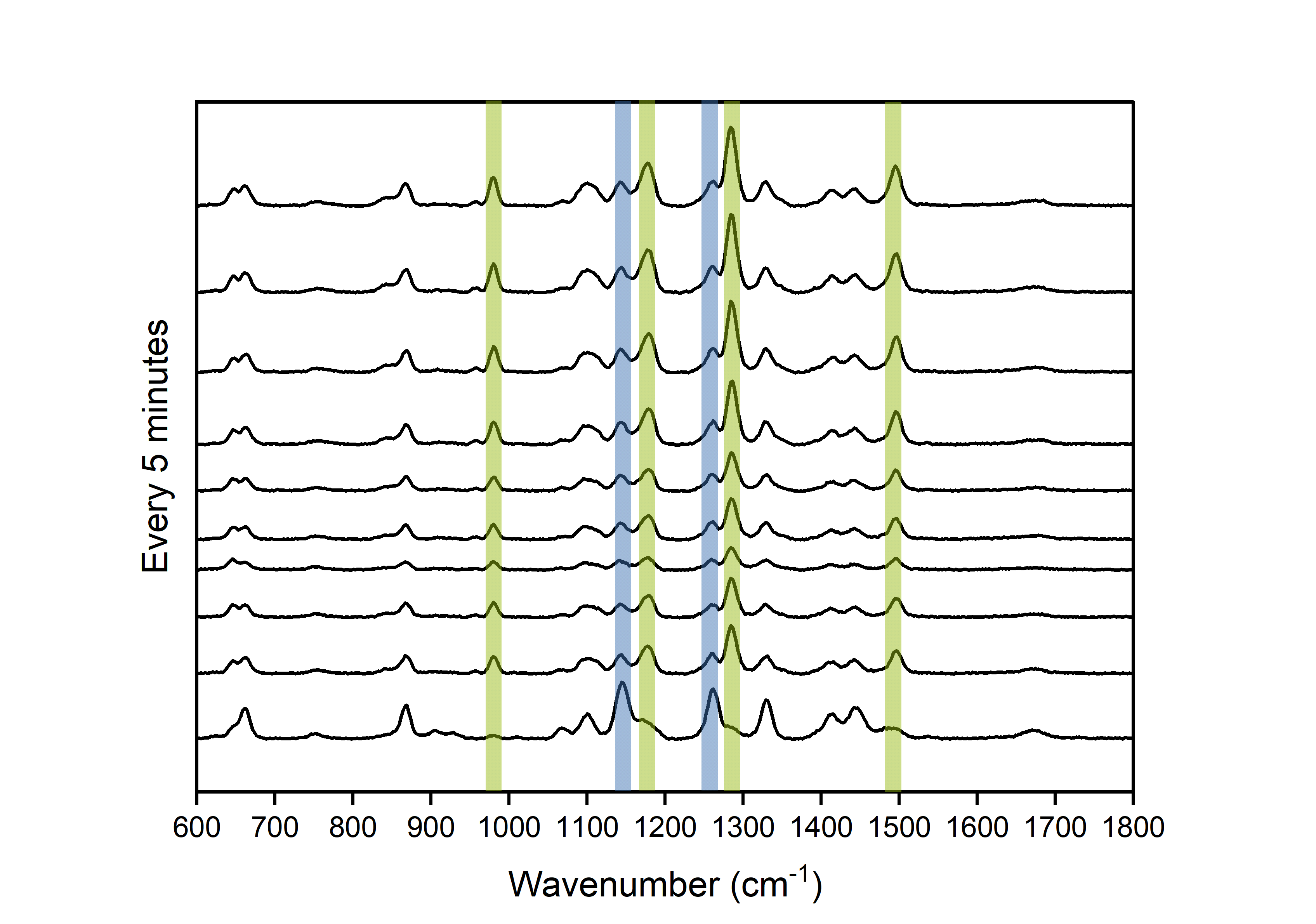


**Figure S12**: Time-resolved Raman spectra measured during the synthesis of zeolitic imidazolate framework, ZIF‑6.


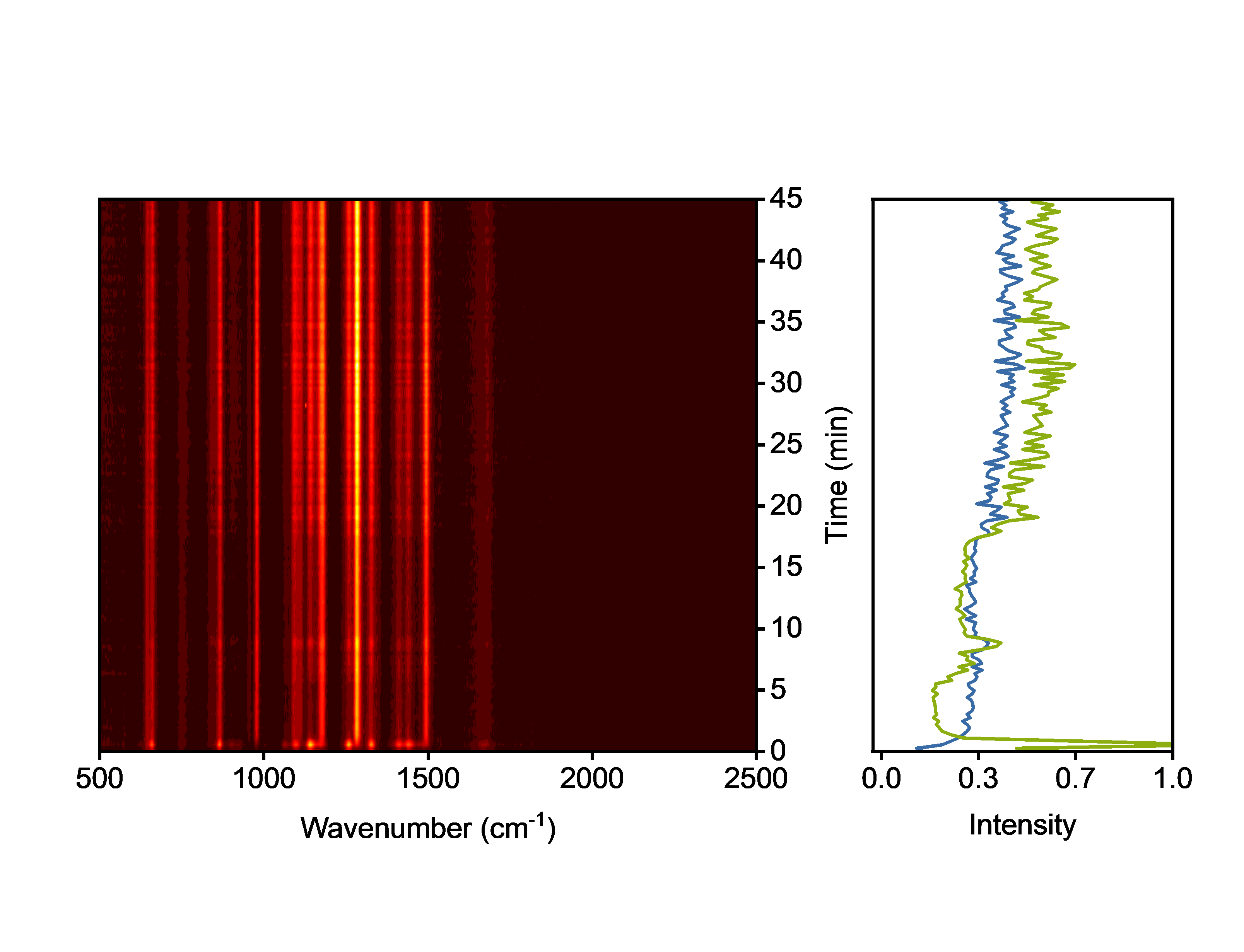


Figure S13: Duplicated synthesis of zeolitic imidazolate framework (ZIF-6) under mechanochemical conditions in the RAM. The same conditions were used to replicate the reaction. This graph presents the full *in situ* Raman spectrum, highlighting at 982 cm^‑1^, 1181 cm^‑1^,1286 cm^‑1^, 1497 cm^‑1^ (blue) the increasing intensity and at 1146 cm^‑1^, 1259 cm^‑1^ (green) the decreasing intensity over time.

1. **Glaser Coupling**


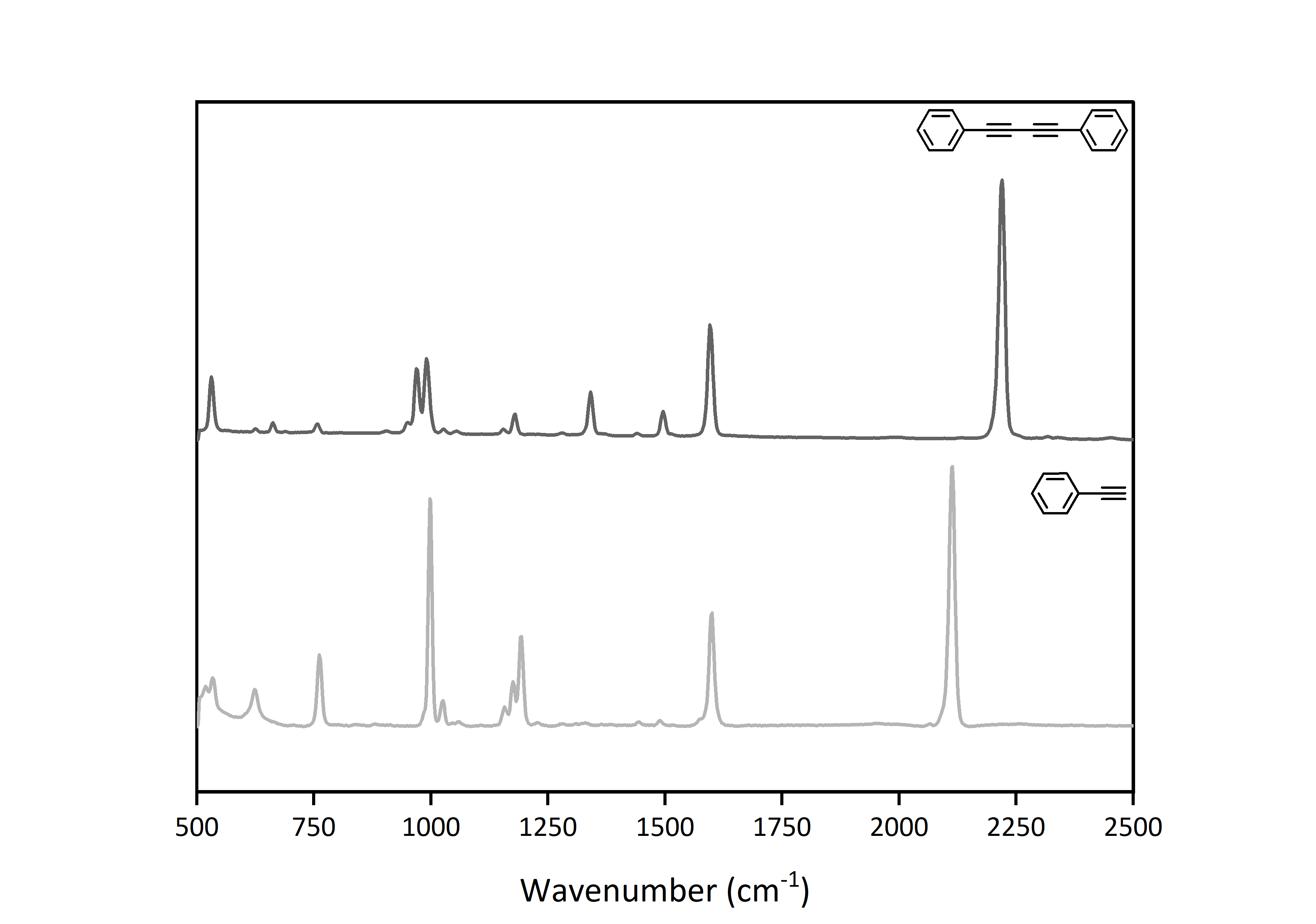


**Figure S14:** Ex situ spectrum of 1,4-diphenylbutadiyne.

The spectrum of 1,4-diphenylbutadiyne is shown at the top, followed by that of phenylacetylene.


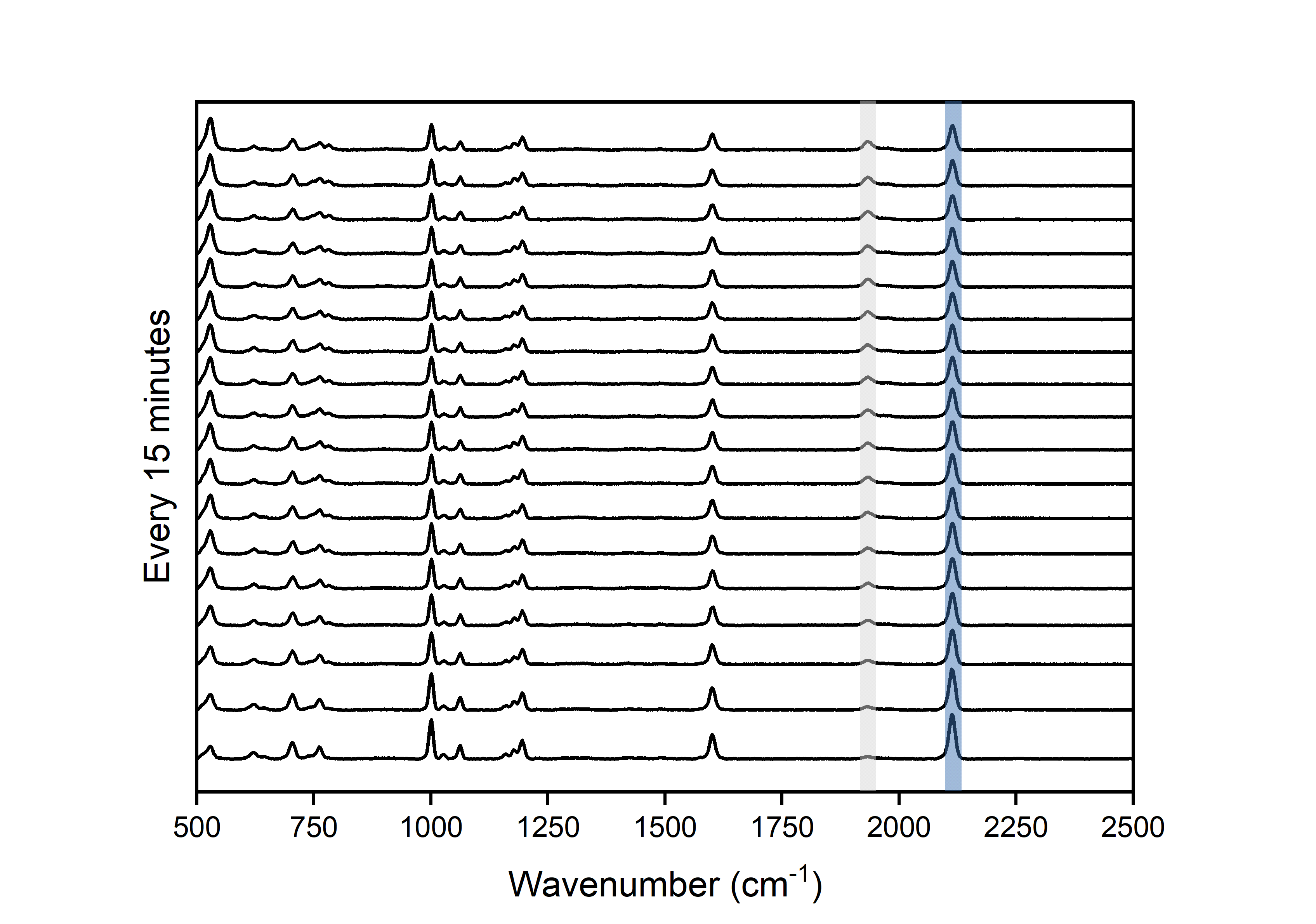


**Figure S15**: Time-resolved Raman spectra measured during the synthesis of 1,4-diphenyl-1,3-diyne.


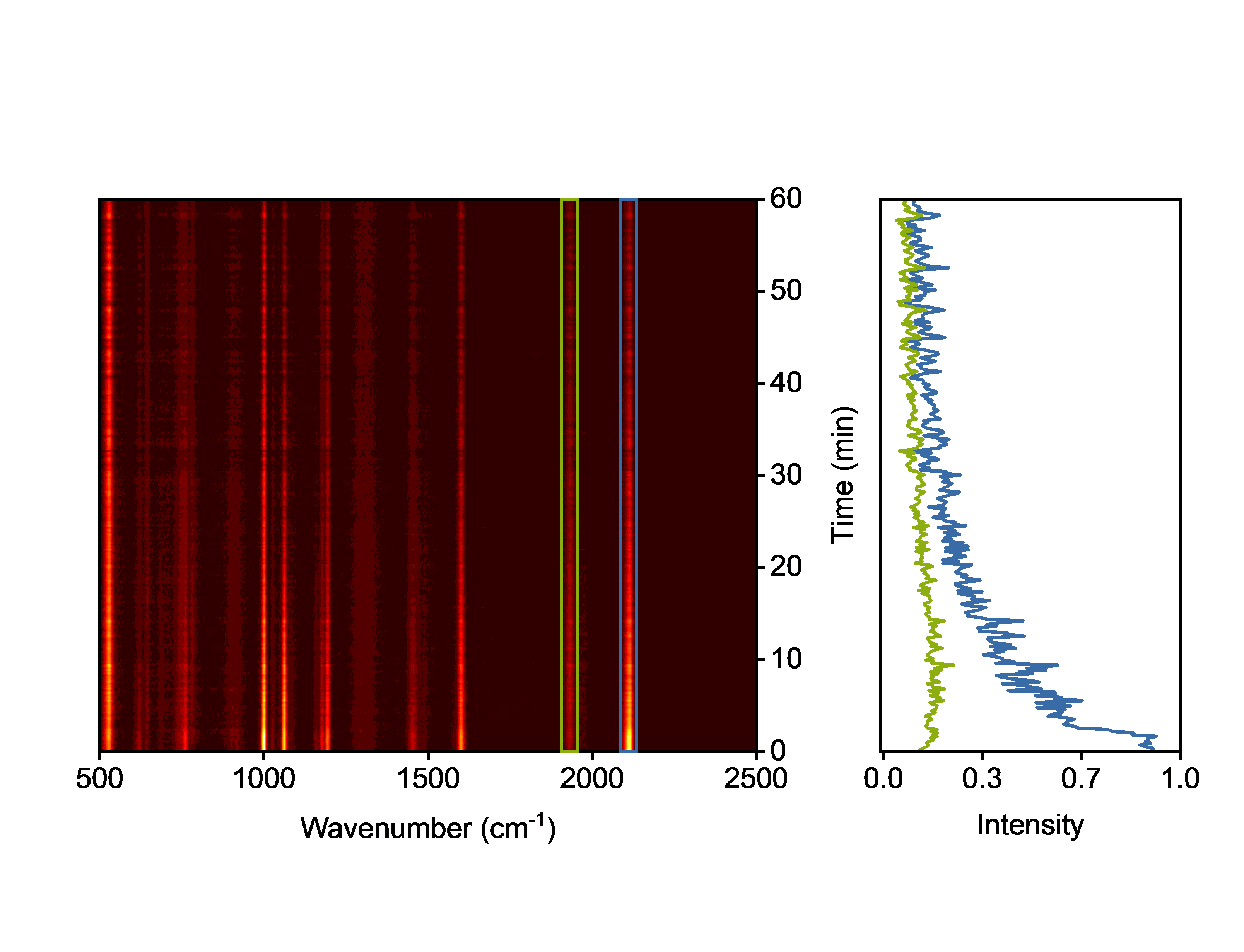


Figure S16: Duplicated synthesis of 1,4-diphenylbuta-1,3-diyne under mechanochemical conditions in the RAM. The same conditions were used to replicate the reaction. This graph presents the full in situ Raman spectrum, with the right section emphasizing the increasing peak intensity at 1935 cm^-1^ (green) corresponding to the Glaser intermediate and the consumption of the substrate (blue) corresponding to phenylacetylene at 2114 cm^‑1^.

# NMR Spectra

**Figure S17**: ^1^H-NMR spectrum of 2-(4-nitrobenzyliden) malononitrile in CDCl_3_, with dibromomethane as an internal standard.

**Figure S18**: ^13^C-NMR spectrum of 2-(4-nitrobenzyliden) malononitrile

in CDCl_3_.

**Figure S19**: ^1^H-NMR spectrum of 2,3-diphenylquinoxaline

in CDCl_3_ with dibromomethane as an internal standard.

**Figure S20**: ^13^C-NMR spectrum of 2,3-diphenylquinoxaline

in CDCl_3_.

# GC-MS

**Figure S21**: GC‑MS spectrum of Knoevenagel condensation

, with dichloromethane (RT = 2.520 min, 3.360 min), 4‑nitrobenzaldehyde (RT = 10.185 min), and 2-(4-nitrobenzylidene) malononitrile (RT = 12.190 min).

# SEM and EDS


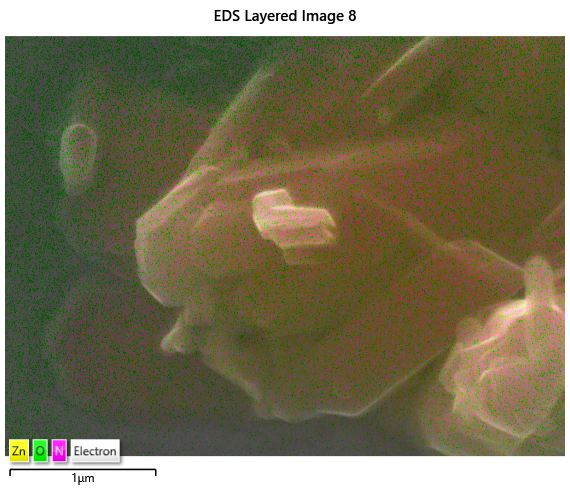

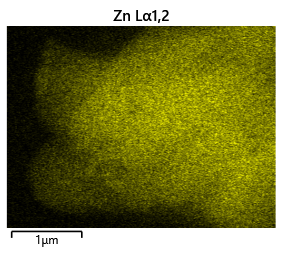

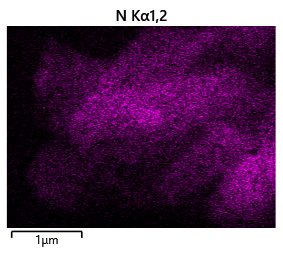

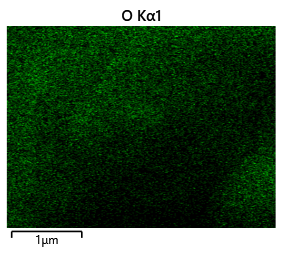


**Figure S22**: EDX mapping of the obtained ZIF‑6 illustrates a spot (on top), with the maps of zinc (yellow), nitrogen (violet), and oxygen (green).


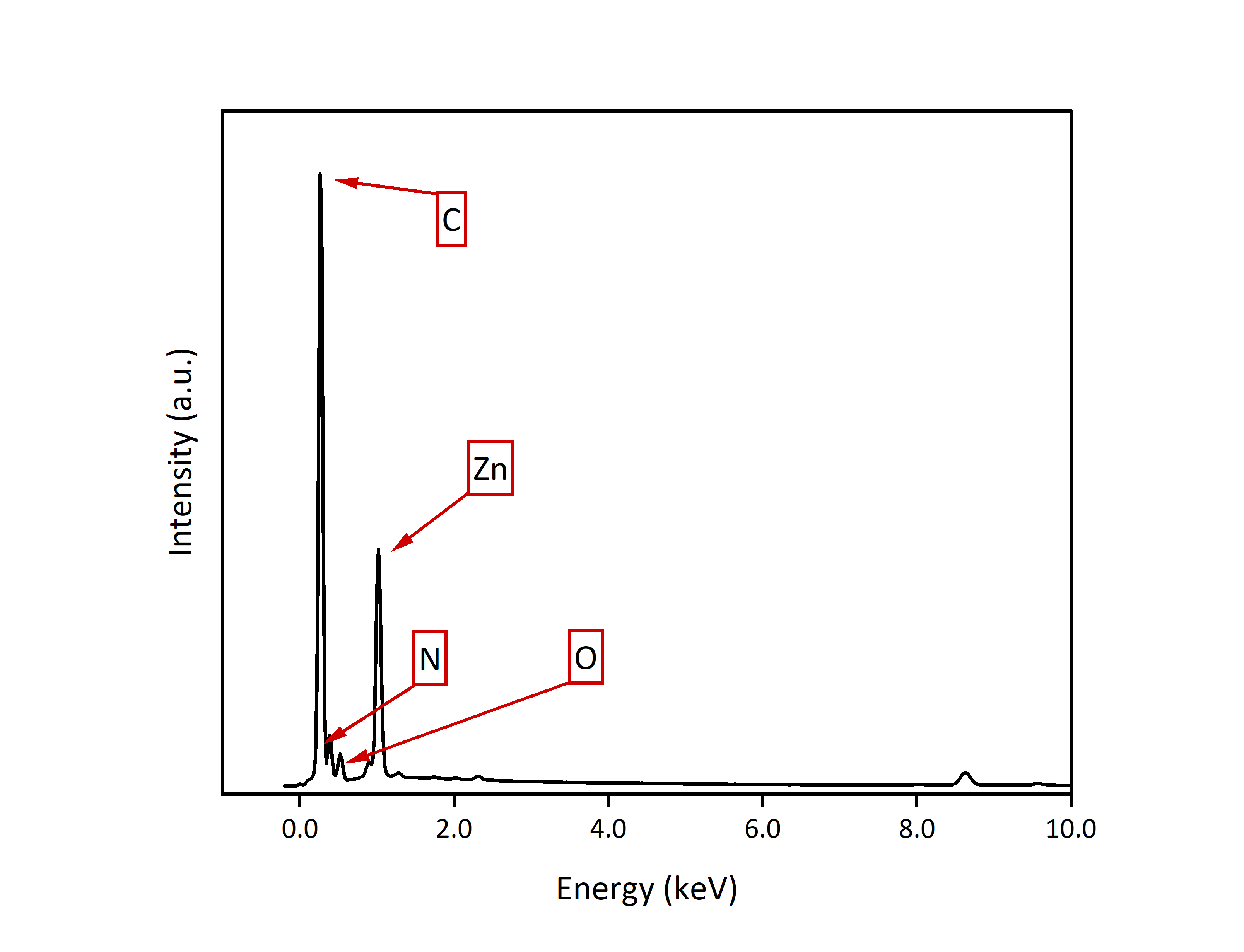


**Figure S23**: EDX spectrum of the obtained ZIF-6 illustrates carbon, nitrogen, oxygen, and zinc.

**Table S5**: EDX elemental composition of the obtained ZIF‑6.

|  | **C (Wt%)** | **N (Wt%)** | **O (Wt%)** | **Zn (Wt%)** |
| --- | --- | --- | --- | --- |
| **Obtained ZIF-6** | 61.52 | 19.51 | 2.59 | 16.20 |

# Literature Comparison

| **Reference** | **Reaction Type** | **Accelaration** | **Reaction Time** | **Filling Degree** | **Operational Window** |
| --- | --- | --- | --- | --- | --- |
| Spula *et al.^[1]^* | Photomechanochemistry | 60 g | 180 min | 12% | Yes |
| Gonnet *et al.^[2]^* | Metal-catalyzed organic reaction | 30-90 g | 30-60 min | 80% | Yes |
| Effaty *et al.^[3]^* | Catalysis | 90 g | 180 min | 10% | No |
| Lennox *et al.^[4]^* | Direct mechanocatalysis | 90 g | 60 min | 30% | No |
| Michalchuk *et al.^[5]^* | Co-crystalization | 50g, 100g | 30 min | 19% | Yes |
| Wohlgemuth *et al.^[6]^* | Direct mechanocatalysis | 60-90 g | 60 min | 7% | Yes |
| Vugrin *et al.^[7]^* | Organic | 80-100g | 120 min | 70-80% | No |
| Hamzehpoor *et al.^[8]^* | Material science | 90g | 180 min | 21% | No |
| Titi *et al.^[9]^* | Material science | 30g | 30 min | 8% | Yes |
| Gonnet *et al.^[10]^* | Organic | 60g, 80g, 100g | 10-120 min | 35% | Yes |

#

# Supporting References

[1] C. Spula, P. M. Preuß, L. Borchardt, S. Grätz, *Solid-State Photochemistry in Resonant Acoustic Mixers*, **2025**.

[2] L. Gonnet, C. B. Lennox, J.-L. Do, I. Malvestiti, S. G. Koenig, K. Nagapudi, T. Friščić, *Angewandte Chemie (International ed. in English)* **2022**, *61*, e202115030.

[3] F. Effaty, L. Gonnet, S. G. Koenig, K. Nagapudi, X. Ottenwaelder, T. Friščić, *Chemical communications (Cambridge, England)* **2023**, *59*, 1010–1013.

[4] C. B. Lennox, T. H. Borchers, L. Gonnet, C. J. Barrett, S. G. Koenig, K. Nagapudi, T. Friščić, *Chemical science* **2023**, *14*, 7475–7481.

[5] A. A. L. Michalchuk, K. S. Hope, S. R. Kennedy, M. V. Blanco, E. V. Boldyreva, C. R. Pulham, *Chemical communications (Cambridge, England)* **2018**, *54*, 4033–4036.

[6] M. Wohlgemuth, S. Schmidt, M. Mayer, W. Pickhardt, S. Grätz, L. Borchardt, *Chemistry (Weinheim an der Bergstrasse, Germany)* **2023**, *29*, e202301714.

[7] L. Vugrin, C. Chatzigiannis, E. Colacino, I. Halasz, *RSC Mechanochem.* **2025**.

[8] E. Hamzehpoor, F. Effaty, T. H. Borchers, R. S. Stein, A. Wahrhaftig‐Lewis, X. Ottenwaelder, T. Friščić, D. F. Perepichka, *Angewandte Chemie* **2024**, *136*.

[9] H. M. Titi, J.-L. Do, A. J. Howarth, K. Nagapudi, T. Friščić, *Chemical science* **2020**, *11*, 7578–7584.

[10] L. Gonnet, C. Lennox, T. Borchers, M. Askari, A. Wahrhaftig-Lewis, S. Koenig, K. Nagapudi, T. Friscic, *Rapid, Scalable Buchwald-Hartwig Amination by Resonant Acoustic Mixing (RAM): Establishing Parameters for RAM Reaction Design*, **2024**.
